# COVID-19 vaccination strategies in settings with limited rollout capacity: a mathematical modelling case study in Sierra Leone

**DOI:** 10.1186/s12889-023-17374-0

**Published:** 2023-12-11

**Authors:** Gizem Mayis Bilgin, Kamalini Lokuge, Ernest Jabbie, Syarifah Liza Munira, Kathryn Glass

**Affiliations:** 1 National Centre for Epidemiology and Population Health, The Australian National University, Canberra, Australia; 2 Ministry of Health and Sanitation, Freetown, Sierra Leone

**Keywords:** COVID-19, SARS-CoV-2, Mathematical model, Vaccination, Vaccine allocation, Vaccine prioritisation

## Abstract

**Background:**

COVID-19 vaccine coverage in low- and middle-income countries continues to be challenging. As supplies increase, coverage is increasingly becoming determined by rollout capacity.

**Methods:**

We developed a deterministic compartmental model of COVID-19 transmission to explore how age-, risk-, and dose-specific vaccine prioritisation strategies can minimise severe outcomes of COVID-19 in Sierra Leone.

**Results:**

Prioritising booster doses to older adults and adults with comorbidities could reduce the incidence of severe disease by 23% and deaths by 34% compared to the use of these doses as primary doses for all adults. Providing a booster dose to pregnant women who present to antenatal care could prevent 38% of neonatal deaths associated with COVID-19 infection during pregnancy. The vaccination of children is not justified unless there is sufficient supply to not affect doses delivered to adults.

**Conclusions:**

Our paper supports current WHO SAGE vaccine prioritisation guidelines (released January 2022). Individuals who are at the highest risk of developing severe outcomes should be prioritised, and opportunistic vaccination strategies considered in settings with limited rollout capacity.

**Supplementary Information:**

The online version contains supplementary material available at 10.1186/s12889-023-17374-0.

## Background

The continued emergence of new COVID-19 variants has shifted the focus of global response strategies from elimination of COVID-19 to the minimisation of severe disease [1]. The global distribution of COVID-19 vaccines has not been equitable [2]. As vaccine supplies in low- and middle-income countries (LMIC) begin to increase, vaccine coverage rates are becoming determined by the capacity of countries to rollout vaccine programmes [3].

COVID-19 vaccine rollouts have been modelled extensively in high-income countries (HIC), published literature in LMIC has been slower to emerge [4]. Vaccine prioritisation strategies from HIC cannot be generalised to LMIC due to differences in age demographics, contact patterns, and seroprevalence between these settings [5]. Many HIC studies assume a stable vaccine supply and sufficient healthcare workforce for reaching universal coverage; these assumptions do not necessarily apply to LMIC.

There have been two main approaches to vaccine prioritisation: directly vaccinating those at highest risk of developing severe outcomes or vaccinating those who contribute most to transmission [4]. Modelling of earlier COVID-19 variants demonstrated that the first approach was justified when supply was low (< 20% population coverage), but the second approach was more beneficial with higher supplies of COVID-19 vaccines [5]. However, with the emergence of Omicron, vaccine prioritisation has been influenced more by vaccine-derived protection against severe outcomes than the impact of vaccines on transmission. The latest World Health Organization Strategic Advisory Group of Experts on Immunization (SAGE) roadmap for prioritising COVID-19 vaccines (released January 2022) advocates for prioritising primary and booster doses to higher risk groups over primary doses to lower risk groups [3]. The roadmap identifies older adults for highest priority use, followed by adults with comorbidities and pregnant individuals for high priority use.

This paper explores the impact of vaccine prioritisation strategies in Sierra Leone, presented as a case study of a low-income setting with limited rollout capacity. We examine the benefits of age-, risk- and dose-specific vaccine prioritisation strategies. We take a novel approach in estimating the benefits of the opportunistic vaccination of pregnant women who present to antenatal care.

## Methods

### COVID-19 transmission model

**Fig. 1:**
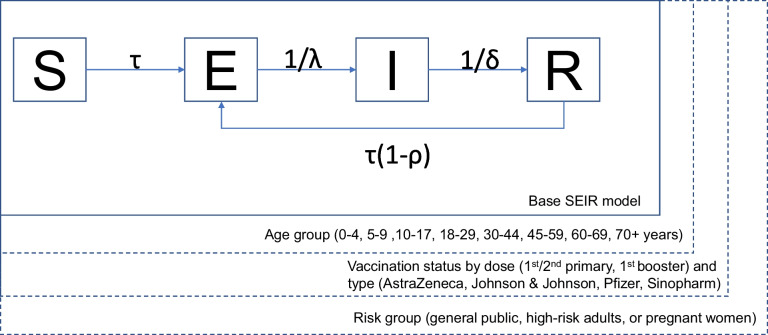
Schematic for model structure including the progression of individuals between Susceptible (S), Exposed (E), Infected (I) and Recovered (R) classes. The parameter $\uptau$ represents the force of transmission, λ the latent period, δ the infectious period, and ρ the effectiveness of infection-derived immunity. See Supplementary Material S1 for the calculation of transmission and Table 1 for the values of all other parameters

We developed a Susceptible-Exposed-Infected-Recovered (SEIR) model for COVID-19 transmission stratified by age, risk group, and vaccination status by dose and type (Fig. 1). Transmission was modulated by vaccine- and infection-derived immunity, both calculated using a daily time step to track the waning of immunity over time. Detail on the model’s configuration and estimation of parameters is provided in the Supplementary Material S1-2.

### Characterisation of study setting

Sierra Leone is a low-income nation in Western Africa [6], with high-levels of non-COVID respiratory disease in all age groups, and limited access to health services. This context makes the mitigation of severe COVID-19 outcomes through vaccination a priority since capacity to implement other control measures is limited.

We extracted vaccine coverage from the Africa Centres for Disease Control and Prevention COVID-19 Vaccine Dashboard with distribution over time from John Hopkins Coronavirus Data Repository [2, 7]. United Nations population estimates for 2022 inform the age structure of our model [8]. Contact patterns were adapted from Prem et al. [9]. The effectiveness of non-pharmaceutical interventions over time was informed by the Oxford COVID-19 Government Response Tracker’s quantification of the strictness of government policies such as school closures, travel bans, and mandated mask-wearing [10]. We fitted the model to our study setting using seroprevalence estimates from March and November 2021 (see Supplementary Material S3).

### Severe outcome projections

We used country-specific severity estimates to project from cases to incidence of severe disease, acute-care bed hospitalisation, and deaths (Table 1) [11]. These wild-type estimates were adjusted by variant- and age-specific multipliers [12, 13]. We calculated years of life lost using United Nations Population Prospect estimates of age-specific life expectancy [8]. We considered vaccine-derived protection against both maternal and pregnancy outcomes when modelling pregnant individuals. See the Supplementary Material S2.4 and S2.5 for further details.

**Table 1:** Model parameters with symbols, descriptions, values, and sources

Parameter	Value	Source(s)
Latent period (λ)	3.71 days (delta) 2.22 days (omicron)	[14] [15]
Infectious period (δ)	10.9 days (delta) 9.87 days (omicron)	[16]
Effectiveness of infection-derived immunity^a^ (ρ)	95%	[17]
Infection acute-care bed hospitalisation rate^b^	0.13% (wild type) 0.26% (delta) 0.20% (omicron)	[11–13]
Infection severity rate^b^	0.27% (wild type) 0.90% (delta) 0.52% (omicron)	
Infection fatality rate^b^	0.034% (wild type) 0.079% (delta) 0.045% (omicron)	
Increased risk of severe outcomes	1.95 (adults with comorbidities) 2.40 (pregnant women)	[18] [19]

^a^Protection against same variant at two weeks, see Supplementary Material S2.3 for effect of immune escape variants and waning over time

^b^Population-level rates, see Supplementary Material S2.4 for age-specific rates

### Existing vaccine coverage in study setting

Sierra Leone has achieved moderate vaccine coverage as of October 2022 – 34.1% single-dose coverage and 25.6% fully vaccinated using AstraZeneca, Johnson & Johnson, Pfizer, and Sinopharm vaccines [2, 7]. The Government of Sierra Leone’s intention is to achieve 51.6% COVID-19 primary schedule coverage, the entire adult population, by the end of 2022 [20]. Booster doses are not yet widely administered in Sierra Leone with 0.1% coverage as of October 2022 [7]. Hence, the initial state of the model included 1,381,537 (single dose vaccine) or 2,763,074 (double dose vaccine) remaining doses to reach the 51.6% target.

### Hypothetical vaccine rollout

We assumed a constant daily rollout capacity of 11,075 doses/day, a conservative estimate slightly lower than the pilot vaccination campaign in Sierra Leone October–November 2021 [20]. We model these doses as distributed in order of priority to eligible age groups until a ‘ceiling’ of vaccine acceptance (1-vaccine hesitancy) is reached. Sierra Leone has reported high but not universal COVID-19 vaccine acceptance – 87.9% (86.2–89.6) [21]. We present results for a range of rollout speeds considering the 2,654 active vaccinators identified by the Sierra Leone Ministry of Health and Sanitation in the Supplementary Material [22].

Our baseline assumption is that future doses will be a single-dose Johnson & Johnson vaccine. The greatest supply of COVID-19 doses in Sierra Leone has been Johnson & Johnson—46.8% Johnson & Johnson compared to 24.9% Pfizer, 18.9% AstraZeneca, 6.4% Sinopharm, and 3.0% Sinovac (as of October 2022) [7]. The Government of Sierra Leone has demonstrated a strong preference for this vaccine, negotiating with major donors, such as the Government of Italy, to change their donations of double-dose vaccines (such as Pfizer) to Johnson & Johnson vaccines for ease of deployment across Sierra Leone [20]. We present sensitivity analysis comparing the prioritisation of a single-dose vaccine (Johnson & Johnson) to a double-dose vaccine (Pfizer) in the Supplementary Material.

### Vaccine prioritisation strategies

We modelled age-, dose- and risk-prioritisation strategies. We examined the benefit of expanding the existing vaccination program to include children, either during or after the rollout of vaccines to eligible adults. We considered scenarios with sufficient vaccine supply for the delivery of a primary schedule to 51.6% (baseline), 60%, 70%, and 75.5% of the population. Note that 75.5% coverage is the highest level of population coverage expected with 88% vaccine acceptance if all aged over five were eligible for COVID-19 vaccination in Sierra Leone.

We considered the prioritisation of two high risk groups – pregnant women and adults with comorbidities – for earlier primary doses or additional booster doses. When considering booster dose delivery, we assumed all adults were eligible three months after their primary schedule, regardless of the vaccine type of their primary schedule. We identified high risk adults as adults with comorbidities aged 30–59 (1.2%) and all adults aged over 60 (4.7%) [20]. We estimated that pregnant women made up 3.3% of the population over a year [23]. We modelled the opportunistic vaccination of pregnant women using data on their presentation to antenatal care from the 2019 Demographic Health Survey in Sierra Leone [23]. We assumed that all pregnant women could be vaccinated within the 4.2 months, the median time to first antenatal care visit for pregnant women. This resulted in an opportunistic vaccination rate of 2,148/day. We did not model the opportunistic vaccination of adults with comorbidities since there is low access to primary care for the management of chronic conditions in Sierra Leone [24, 25]. We present sensitivity analysis for reduced vaccine effectiveness in older adults or adults with comorbidities, and for increased vaccine hesitancy in pregnant women in the Supplementary Material.

## Results

The expansion of COVID-19 vaccine eligibility to children is beneficial provided children are vaccinated after adults (Table 2). Broadening the vaccine rollout to include children and adults concurrently can lead to an increase in the incidence of severe disease in adults due to the reduction in daily capacity for vaccine rollout in adults (given overall vaccination capacity remains constant). Vaccinating children concurrently with adults was especially detrimental with a double-dose vaccine such as Pfizer, increasing overall deaths by 10.1–17.5% (Table S4.1).

**Table 2:** Cumulative outcomes prevented by prioritisation strategies including children 5 to 17 years. Outcomes prevented calculated relative to current adult vaccination program with a population coverage target of 51.6%, with bold text indicating that the intervention leads to increases in an outcome. Note that 75.5% coverage is the maximum coverage possible with eligibility expanded to 5 + years with 88% vaccine acceptance

	**Children (0–4 years)**					**Children (5–17 years)**					**Adults (18 + years)**					**Overall**				
	Cases	Severe disease	Hospitalisations	Deaths	Years of life lost	Cases	Severe disease	Hospitalisations	Deaths	Years of life lost	Cases	Severe disease	Hospitalisations	Deaths	Years of life lost	Cases	Severe disease	Hospitalisations	Deaths	Years of life lost
**Expanding to children concurrently with the adult rollout**																				
51.6%	**3148**	**0**	**39**	**0**	**5**	-85,506	-64	-3859	-7	-343	**65,605**	**1436**	**4595**	**100**	**1184**	-16,752	**1372**	**775**	**93**	**846**
	**0.2%**	**0.3%**	**0.3%**	**0.3%**	**0.3%**	-2.3%	-9.4%	-9.4%	-9.4%	-9.4%	**1.5%**	**7.0%**	**7.0%**	**7.0%**	**7.1%**	-0.2%	**6.5%**	**0.6%**	**6.2%**	**3.8%**
60%	-8561	-1	-92	0	-11	-147,586	-87	-5200	-9	-462	**3664**	**867**	**2856**	**57**	**702**	-152,483	**780**	-2437	**48**	**229**
	-0.7%	-0.6%	-0.6%	-0.6%	-0.6%	-4.0%	-12.6%	-12.6%	-12.6%	-12.6%	**0.1%**	**4.3%**	**4.3%**	**4.0%**	**4.2%**	-1.6%	**3.7%**	-2.0%	**3.2%**	**1.0%**
70%	-39,413	-5	-461	-1	-54	-280,860	-116	-6963	-12	-618	-128,859	**10**	**180**	-6	-18	-449,132	-111	-7244	-18	-690
	-3.1%	-3.0%	-3.0%	-3.0%	-3.0%	-7.6%	-16.9%	-16.9%	-16.9%	-16.9%	-3.0%	**0.0%**	**0.3%**	-0.4%	-0.1%	-4.8%	-0.5%	-5.9%	-1.2%	-3.1%
75.5%	-49,597	-6	-585	-1	-68	-337,962	-126	-7588	-13	-674	-174,788	-259	-672	-25	-243	-562,348	-392	-8845	-39	-984
	-3.9%	-3.8%	-3.8%	-3.8%	-3.8%	-9.1%	-18.4%	-18.4%	-18.4%	-18.4%	-4.1%	-1.3%	-1.0%	-1.8%	-1.4%	-6.1%	-1.8%	-7.2%	-2.6%	-4.4%
**Expanding to children after adult rollout**																				
60%	-11,382	-1	-126	0	-15	-105,324	-50	-2982	-5	-265	-41,161	-237	-669	-20	-212	-157,867	-288	-3777	-26	-491
	-0.9%	-0.8%	-0.8%	-0.8%	-0.8%	-2.8%	-7.2%	-7.2%	-7.2%	-7.2%	-1.0%	-1.2%	-1.0%	-1.4%	-1.3%	-1.7%	-1.4%	-3.1%	-1.7%	-2.2%
70%	-39,527	-5	-463	-1	-54	-266,378	-89	-5316	-9	-472	-139,553	-753	-2249	-60	-653	-445,458	-846	-8028	-70	-1178
	-3.1%	-3.0%	-3.0%	-3.0%	-3.0%	-7.2%	-12.9%	-12.9%	-12.9%	-12.9%	-3.2%	-3.7%	-3.4%	-4.2%	-3.9%	-4.8%	-4.0%	-6.6%	-4.6%	-5.3%
75.5%	-48,559	-6	-573	-1	-67	-337,145	-102	-6124	-10	-543	-170,293	-912	-2741	-72	-789	-555,997	-1020	-9438	-84	-1399
	-3.8%	-3.7%	-3.7%	-3.7%	-3.7%	-9.1%	-14.9%	-14.9%	-14.9%	-14.9%	-4.0%	-4.5%	-4.2%	-5.1%	-4.7%	-6.0%	-4.8%	-7.7%	-5.5%	-6.3%

Providing older adults and adults with comorbidities with a booster dose could reduce the incidence of severe disease by 22.8% and deaths by 34.3% (Table 3). Prioritising these high-risk adults for primary doses only has limited current benefit due to existing high primary coverage in this group. However, adults at highest risk of developing severe outcomes should be prioritised in any future expansions of the vaccination program (Table S4.3). Sensitivity analysis for reduced vaccine effectiveness in older adults and adults with comorbidities also supports the provision of a booster dose – reducing severe disease by 24.1% and deaths by 36.2% (Table S4.7).

**Table 3:** Cumulative outcomes prevented by risk-specific prioritisation strategies. Outcomes prevented calculated relative to current vaccination program with a population coverage target of 51.6% and uniform eligibility

	Incidence	Incidence of severe disease	Hospitalisations	Deaths	Years of life lost^a^	Neonatal deaths
Pregnant women						
**Prioritising within existing rollout capacity**						
25% primary dose only	779 (0.0%)	6 (0.0%)	-11 (0.0%)	1 (0.1%)	9 (0.0%)	-30 (-0.9%)
50% primary dose only	1,142 (0.0%)	7 (0.0%)	-8 (0.0%)	1 (0.1%)	10 (0.0%)	-32 (-1.0%)
75% primary dose only	1,278 (0.0%)	8 (0.0%)	-6 (0.0%)	1 (0.1%)	10 (0.0%)	-32 (-1.0%)
50% primary and booster provision	-24,466 (-0.3%)	-293 (-1.4%)	-2,120 (-1.7%)	1 (0.0%)	-99 (-0.4%)	-1,223 (-38.2%)
**Additional rollout capacity (opportunistic vaccination during antenatal visits)**						
Additional primary doses	-2,591 (0.0%)	-35 (-0.2%)	-168 (-0.1%)	-2 (-0.1%)	-28 (-0.1%)	-30 (-0.9%)
Additional primary and booster doses	-31,022 (-0.3%)	-413 (-1.9%)	-2,547 (-2.1%)	-8 (-0.5%)	-207 (-0.9%)	-1,209 (-37.8%)
Expanding eligibility to high-risk children	-5,754 (-0.1%)	-42 (-0.2%)	-425 (-0.3%)	-3 (-0.2%)	-48 (-0.2%)	-174 (-5.4%)
Adults with comorbidities						
**Prioritising within existing rollout capacity**						
25% primary dose only	1,339 (0.0%)	-69 (-0.3%)	-106 (-0.1%)	-8 (-0.5%)	-72 (-0.3%)	NA
50% primary dose only	3,106 (0.0%)	-90 (-0.4%)	-126 (-0.1%)	-11 (-0.7%)	-95 (-0.4%)	NA
75% primary dose only	4,007 (0.0%)	-93 (-0.4%)	-121 (-0.1%)	-11 (-0.7%)	-98 (-0.4%)	NA
50% primary and booster provision	-46,484 (-0.5%)	-4,829 (-22.8%)	-9,697 (-7.9%)	-520 (-34.3%)	-4,848 (-21.8%)	NA

^a^Years of life lost does not include neonatal deaths due to COVID-19 infection during pregnancy to allow comparison between scenarios where pregnant women are not modelled explicitly. Life expectancy at birth in Sierra Leone is 60.4 years [8]

Prioritising pregnant women within a restricted rollout capacity may not be beneficial due to the delayed vaccination of more vulnerable older adults. However, the opportunistic vaccination of pregnant women who present to antenatal care (outside of a COVID-19 vaccine program) appears beneficial. A catch-up program for unvaccinated pregnant women who present to antenatal care could reduce neonatal deaths by 0.9%. Providing all pregnant women who present to antenatal care with a booster dose could prevent 37.8% of neonatal deaths by boosting vaccine-derived protection against adverse pregnancy outcomes associated with COVID-19 infection.

Interestingly, the speed of rollout did not affect the cumulative incidence of severe outcomes during the continued circulation of COVID-19 (Figure S4.2B). Additionally, due to ongoing transmission, the timing of vaccination relative to outbreaks did not create a meaningful difference in cumulative outcomes (Figure S4.2A).

## Discussion

Vaccinating individuals who are at the highest risk of severe outcomes remains the priority as COVID-19 continues to circulate in settings with low supply and limited rollout capacity. Prioritising booster doses to high-risk groups is justified – reducing severe disease (23%) and death (34%) in older adults and adults with comorbidities. Our results also support the opportunistic vaccination of high-risk groups such as pregnant women who present to antenatal care. Providing pregnant women with booster doses could prevent 38% of neonatal deaths associated with adverse pregnancy outcomes after COVID-19 infection during pregnancy. Vaccinating children is beneficial when vaccine supplies are great enough that vaccination of children does not divert doses away from more vulnerable adults.

Our findings strongly support current World Health Organization SAGE and Africa Centers for Disease Control and Prevention prioritisation guidelines [3, 26]. Prioritising primary and booster doses to high-risk groups yields higher reductions in severe disease than using vaccine supply to increase primary dose coverage in the general population. Our findings are consistent with previous modelling studies which support prioritising vulnerable adults (by age or comorbidity) when vaccine rollout is slow, if vaccine effectiveness against acquisition is low, and/or if community transmission is high [27–32]. Our study contributes to a gap in single-country studies of vaccine prioritisation in low-income settings [4]. Notable examples modelling COVID-19 vaccination strategies in low-income countries include work from Ghana [33] and Madagascar [34].

In this study, we present the first results for the opportunistic provision of booster doses to pregnant women who present to antenatal care. COVID-19 vaccination during pregnancy has been assessed to pose no increased risk of adverse pregnancy outcomes [35], while COVID-19 infection during pregnancy has been linked to poorer maternal and pregnancy outcomes [19]. The rollout of COVID-19 vaccines to adults has been hindered by the lack of prior experience and infrastructure for adult vaccination in LMIC [36]. High uptake of antenatal care by pregnant women in Sierra Leone makes these visits a prime target for opportunistic vaccination [23].

Our study has several limitations. Firstly, there is considerable uncertainty surrounding COVID-19 characteristics as new variants continue to emerge, and the morbidity associated with ‘long COVID’ has not yet been quantified. Secondly, we model pregnant women and adults with comorbidities as homogenous groups. This assumption is reasonable for modelling the benefits of vaccination, but program planning will need to be mindful of this heterogeneity. COVID-19 infection and associated hospitalisation appears more common later in pregnancy [37], and not all comorbidities present equal risk of severe outcomes with COVID-19 [38]. Thirdly, we assumed equal vaccine-derived protection against adverse pregnancy outcomes as protection for pregnant women against severe disease. Future modelling studies would benefit from data on the effectiveness of vaccines in preventing adverse pregnancy outcomes due to COVID-19 infection. Fourthly, our model uses COVID-19 campaign estimates for the speed of vaccine rollout. As COVID-19 becomes endemic, a more sustainable speed of COVID-19 rollout must be quantified to not impair other public health priorities. Finally, our model does not include maternally derived immunity in infants whose mothers were vaccinated. COVID-19 vaccines in pregnancy may provide additional benefits in the protection or young infants who are at high risk of severe disease.

## Conclusions

This paper supports the prioritisation of booster doses to those at highest risk of developing severe outcomes. Older adults and adults with comorbidities should receive priority access within campaign rollout capacity. Vaccine eligibility should be expanded to children after all adults willing to be vaccinated receive their primary schedule. The opportunistic vaccination with additional vaccine supplies of high-risk groups, such as pregnant women, should be considered in settings with limited rollout capacity. Future studies must consider the sustainability and benefits of ongoing COVID-19 vaccination in settings with restricted health workforce capacity.

### Supplementary Information

**Additional file 1: S1.** Model Structure. **S1.1.** Modelling transmission. **S1.2.** Fitting to the basic reproduction number. **S1.3.** Tracking the effective reproduction number. **S1.4.** Modification of severe outcome projections with high-risk group. **S2.** Parameter Estimates. **S2.1.** Parameters associated with transmission. **S2.2.** Vaccine effectiveness. **S2.3.** Infection-derived immunity. **S2.4.** Severe outcome projections. **S2.5.** Characterising high-risk groups. **S3.** Model Fit. **S4.** Sensitivity Analysis. **S4.1.** Outbreak of a new immune-escape variant. **S4.2.** Prioritisation of a double-dose vaccine. **S4.3.** Prioritisation of a larger number of doses. **S4.4.** Increased vaccine hesitancy in pregnant women. **S4.5.** Reduced vaccine effectiveness in older adults. **S4.6.** Reduced vaccine effectiveness in older adults and adults with comorbidities. **S4.7.** Increased or decreased risk of high-risk groups. **S4.8.** Influence of pre-existing infection-derived immunity.

## Data Availability

We collated all data from publicly available data sources. All data and code can be viewed on our GitHub: https://github.com/gizembilgin/vaccine_prioritisation_SLE.

## Abbreviations

COVID-19: Coronavirus disease 2019
HIC: High-Income Countries
LMIC: Low- and Middle-Income Countries
SAGE: Strategic Advisory Group of Experts on Immunization
SEIR: Susceptible-Exposed-Infected-Recovered
WHO: World Health Organization
