# Supplementary material for: COVID-19 vaccination strategies in settings with limited rollout capacity: a mathematical modelling case study in Sierra Leone
Source: BMC Public Health. 2023 Dec 11;23:2466. doi: 10.1186/s12889-023-17374-0 (PMC10712073; doi:10.1186/s12889-023-17374-0)
Supplement: Supplementary file 1 — Additional file 1: S1. Model Structure. S1.1. Modelling transmission. S1.2. Fitting to the basic reproduction number. S1.3. Tracking the effective reproduction number. S1.4. Modification of severe outcome projections with high-risk group. S2. Parameter Estimates. S2.1. Parameters associated with transmission. S2.2. Vaccine effectiveness. S2.3. Infection-derived immunity. S2.4. Severe outcome projections. S2.5. Characterising high-risk groups. S3. Model Fit. S4. Sensitivity Analysis. S4.1. Outbreak of a new immune-escape variant. S4.2. Prioritisation of a double-dose vaccine. S4.3. Prioritisation of a larger number of doses. S4.4. Increased vaccine hesitancy in pregnant women. S4.5. Reduced vaccine effectiveness in older adults. S4.6. Reduced vaccine effectiveness in older adults and adults with comorbidities. S4.7. Increased or decreased risk of high-risk groups. S4.8. Influence of pre-existing infection-derived immunity. [file 12889_2023_17374_MOESM1_ESM.docx]

**COVID-19 vaccination strategies in settings with limited rollout capacity: a mathematical modelling case study in Sierra Leone**

*Supplementary Material*

**Table of Contents**

[**S1. Model Structure** 2](#_Toc146188564)

[**S1.1 Modelling transmission** 2](#_Toc146188565)

[**S1.2 Fitting to the basic reproduction number** 2](#_Toc146188566)

[**S1.3 Tracking the effective reproduction number** 2](#_Toc146188567)

[**S1.4 Modification of severe outcome projections with high-risk group** 3](#_Toc146188568)

[**S2. Parameter Estimates** 4](#_Toc146188569)

[**S2.1 Parameters associated with transmission** 4](#_Toc146188570)

[**S2.2 Vaccine effectiveness** 4](#_Toc146188571)

[**S2.3 Infection-derived immunity** 7](#_Toc146188572)

[**S2.4 Severe outcome projections** 9](#_Toc146188573)

[**S2.5 Characterising high-risk groups** 10](#_Toc146188574)

[**S3. Model Fit** 12](#_Toc146188575)

[**S4. Sensitivity Analysis** 15](#_Toc146188576)

[**S4.1 Outbreak of a new immune-escape variant** 15](#_Toc146188577)

[**S4.2 Prioritisation of a double-dose vaccine** 17](#_Toc146188578)

[**S4.3 Prioritisation of a larger number of doses** 19](#_Toc146188579)

[**S4.4 Increased vaccine hesitancy in pregnant women** 20](#_Toc146188580)

[**S4.5 Reduced vaccine effectiveness in older adults** 21](#_Toc146188581)

[**S4.6 Reduced vaccine effectiveness in older adults and adults with comorbidities** 24](#_Toc146188582)

[**S4.7 Increased or decreased risk of high-risk groups** 25](#_Toc146188583)

[**S4.8 Influence of pre-existing infection-derived immunity** 28](#_Toc146188584)

[**Reference List** 32](#_Toc146188585)

# **S1. Model Structure**

## **S1.1 Modelling transmission**

Transmission (τ) was modelled as a product of daily contacts between age-classes (C), the proportion of their contacts who are infected (I/N), effectiveness of non-pharmaceutical interventions (NPI), and a scaling factor (β) used to fit transmission to the basic reproduction number of the circulating strain. Hence, the rate of daily transmission in the $i$’th age group was defined as the sum of possible transmissions to them from individuals of age classes ‘$j$’,

$$\tau\left( i \right)= \beta*(1-NPI)*\sum_{j} C_{i,j}*\frac{I\left( j \right)}{N\left( j \right)}.$$

Estimates for all basic transmission parameters were kept constant between age groups. Differences in transmission dynamics arose from age-specific differences in contact patterns, vaccine coverage, and infection-derived immunity.

We modelled vaccination using a ‘leaky vaccine’ approach in which all vaccinated individuals have a reduced probability of infection by days since vaccination,

$$\tau\left( i \right)=\tau\left( i \right)*\left( 1-{VE}_{dose,type}[days] \right).$$

For individuals in the recovered class, transmission was also modulated by the effectiveness of infection-derived immunity by time since previous infection:

$$\tau\left( i \right)= \tau\left( i \right)*\left( 1-\rho[days] \right).$$

## **S1.2 Fitting to the basic reproduction number**

The basic reproduction number $(R_{0})$ represents the average number of secondary infections resulting from one case interacting with an entirely susceptible population in the absence of control measures. We calculated $R_{0}$ as the spectral radius the next-generation matrix (NGM):

$$R_{0}=\rho\left( NGM \right)$$

$$NGM=\tilde{C}*diag\left\{ \beta*\frac{1}{\delta} \right\}$$

$$\tilde{C}_{i,j}=\frac{N(i)}{N(j)}*C_{i,j}$$

Where $\tilde{C}$ represents the contact matrix weighted by population distribution, see [1-5]. We fitted the model to the $R_{0}$ of the circulating strain by adjusting the scaling factor $\beta$. We used an $R_{0}$ estimate of 2.79 for wild-type [6], and 5.08 for Delta [7]. We assumed that Omicron has an equivalent $R_{0}$ to Delta but is an immune-escape variant (see S2.3).

## **S1.3 Tracking the effective reproduction number**

We included the impact of non-pharmaceutical interventions $(NPI)$, infection-derived immunity $(\rho)$, and vaccine-derived immunity when calculating the effective reproduction number $(R_{eff})$**:**

$$R_{eff}=\rho\left( NGM \right)$$

$${NGM}_{Reff}={NGM}_{R0}*\left( 1-NPI \right)*(1-immunity)$$

$(1-immunity)= diag\left\{ \sum_{S,E,I,R} \left\{ \begin{aligned} \left( 1-\rho\right)*\left( 1-\sum_{type,dose} vaccine coverage*vaccine effectiveness \right), R \\ \left( 1-\sum_{type,dose} vaccine coverage*vaccine effectiveness \right), SEI \end{aligned} \right. \right\}$

## **S1.4 Modification of severe outcome projections with high-risk group**

When including a high-risk group, we adjusted the incidence of severe outcomes in the general population based on the increased risk in the high-risk group ($RR)$ given the equations:

$$\frac{C_{RISK}}{P_{RISK}} \approx RR*\frac{C_{GENERAL POP}}{P_{GENERAL POP}}$$

$$C_{RISK}\approx RR*\frac{C_{GENERAL POP}*P_{RISK}}{P_{GENERAL POP}}$$

$$C_{GENERAL POP}\approx\frac{C_{RISK}*P_{GENERAL POP}}{P_{RISK}*RR}$$

Solving simultaneous equations:

$$incidence rate \left( IR \right)=\frac{cases \left( C \right)}{population \left( P \right)}=\frac{C_{RISK}+ C_{GENERAL POP}}{P}$$

$$IR =\frac{RR*\frac{P_{RISK}*C_{GENERAL POP}}{P_{GENERAL POP}}+ C_{GENERAL POP}}{P}$$

$$IR =\frac{\left( RR*\frac{P_{RISK}}{P_{GENERAL POP}}+1 \right)* C_{GENERAL POP}}{P}$$

$$C_{GENERAL POP}=\frac{IR*P}{RR*\frac{P_{RISK}}{P_{GENERAL POP}}+1}$$

$$C_{RISK}=\frac{IR*P}{\frac{P_{GENERAL POP}}{P_{RISK}*RR}+1}$$

Resultant equations:

$${IR}_{GENERAL POP}=\frac{C_{GENERAL POP}}{P_{GENERAL POP}}=\frac{\frac{IR*P}{\frac{{RR*P}_{RISK}}{P_{GENERAL POP}}+1}}{P_{GENERAL POP}}$$

$${IR}_{RISK}=\frac{C_{RISK}}{P_{RISK}}=\frac{\frac{IR*P}{\frac{P_{GENERAL POP}}{P_{RISK}*RR}+1}}{P_{RISK}}$$

# **S2. Parameter Estimates**

## **S2.1 Parameters associated with transmission**

**Table S2.1** Model parameters with symbols, descriptions, values, and sources.

| **Symbol** | **Description** | **Value** | **Source** |
| --- | --- | --- | --- |
| $\gamma$ | Proportion of infected individuals who are symptomatic | 29% (0-9 years)  21% (10-19 years)  27% (20-29 years)  33% (30-39 years)  40% (40-49 years)  49% (50-59 years)  63% (60-69 years)  69% (70+ years) | [8] |

**Table S2.2** Daily age-specific contact patterns for Sierra Leone, adapted from [9]

|  | **Age group of contact** | | | | | | | | **Total daily contacts** |
| --- | --- | --- | --- | --- | --- | --- | --- | --- | --- |
| **Age group** | **0 to 4** | **5 to 9** | **10 to 17** | **18 to 29** | **30 to 44** | **45 to 59** | **60 to 69** | **70+** |  |
| 0 to 4 | 2.5 | 1.9 | 1.6 | 2.7 | 3.9 | 2.3 | 1.0 | 0.4 | 16.5 |
| 5 to 9 | 2.4 | 9.4 | 2.7 | 2.1 | 4.4 | 2.1 | 0.9 | 0.3 | 24.3 |
| 10 to 17 | 0.8 | 2.5 | 11.1 | 3.7 | 3.3 | 1.9 | 0.5 | 0.3 | 24.2 |
| 18 to 29 | 0.9 | 0.7 | 3.0 | 5.9 | 3.6 | 2.2 | 0.4 | 0.2 | 16.9 |
| 30 to 44 | 1.0 | 1.2 | 1.2 | 2.3 | 5.2 | 2.8 | 0.6 | 0.2 | 14.6 |
| 45 to 59 | 0.8 | 0.8 | 1.1 | 1.8 | 3.5 | 2.9 | 0.5 | 0.1 | 11.6 |
| 60 to 69 | 1.1 | 1.1 | 0.9 | 1.2 | 2.4 | 1.9 | 0.5 | 0.1 | 9.4 |
| 70+ | 0.4 | 0.7 | 0.9 | 0.5 | 0.8 | 0.7 | 0.3 | 0.2 | 4.5 |

## **S2.2 Vaccine effectiveness**

Our model includes vaccine effectiveness (VE) against infection, severe disease, and death. We modelled individuals as gaining immunity 21 days after their first dose, and 14 days after all subsequent doses. We estimated VE by vaccine type, dose, outcome, and circulating strain using the ongoing systematic review jointly conducted by the International Vaccine Access Center and World Health Organisation [10]. This systematic review does not provide pooled estimates due to differences in study population and length of follow-up. We examined the studies which met the inclusion criteria of the systematic review and selected estimates that were most representative of a general population, i.e., not limited to subgroups such as healthcare workers. We checked that the chosen estimates aligned broadly with the other estimates provided in the systematic review. When multiple time intervals of follow-up were reported, we chose the earliest interval to minimise the effects of waning immunity since waning was included using a distribution in a later step. We assumed VE against Omicron sub-lineages to be equal (supported to date by [11, 12]).

Where an estimate of VE was not available, an estimate was imputed from the most relevant available estimate in the following order: dose, outcome, strain. The ratio of effectiveness between dose one and two was kept consistent between strains and vaccine types but varied by outcome. The ratio of effectiveness between dose one and two was 0.70 against any infection (standard deviation (σ) 0.15), 0.60 against symptomatic infection (σ 0.11), 0.83 against severe disease (σ 0.13), and 0.89 against death (σ 0.05). VE against death and VE against severe outcomes aligned broadly (ratio 1.02, σ 0.02). VE against infection aligned with VE against symptomatic disease for the Delta variant (ratio 1.03, σ 0.11), but not Omicron (ratio 0.53, σ 0.05). The reduction in effectiveness against the Delta and Omicron variants varied by outcome: any infection (0.45, σ 0.02), severe disease (0.62, σ 0.16), and symptomatic disease (0.71, σ 0.11).

We estimated the effectiveness of a booster dose by averaging across estimates of heterologous combinations as expected in our study setting. That is, to estimate the effectiveness of Pfizer booster doses we considered VE studies where Pfizer boosters were delivered to individuals who had received AstraZeneca, Sinopharm, or Pfizer primary schedules (Figure S2.3). Similar protection against severe outcomes was provided by a Pfizer booster dose across all heterologous combinations. There was insufficient data to consider heterologous combinations with a Johnson & Johnson booster. Hence, we assumed all individuals had protection equal to a Johnson & Johnson primary schedule + Johnson & Johnson booster dose regardless of their primary schedule.


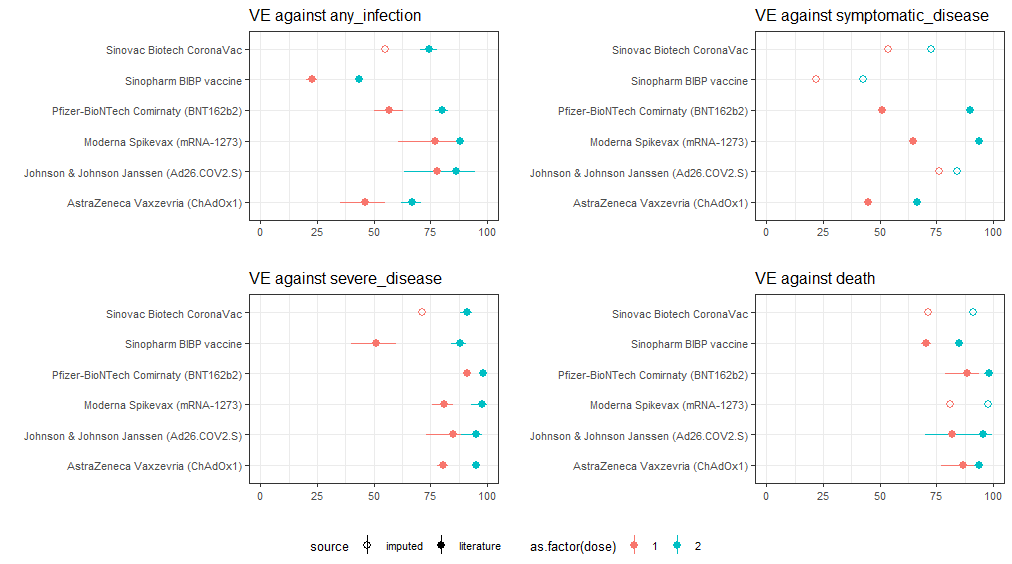
 **Figure S2.1** Vaccine effectiveness estimates against the Delta variant [13-23] as selected from [10]


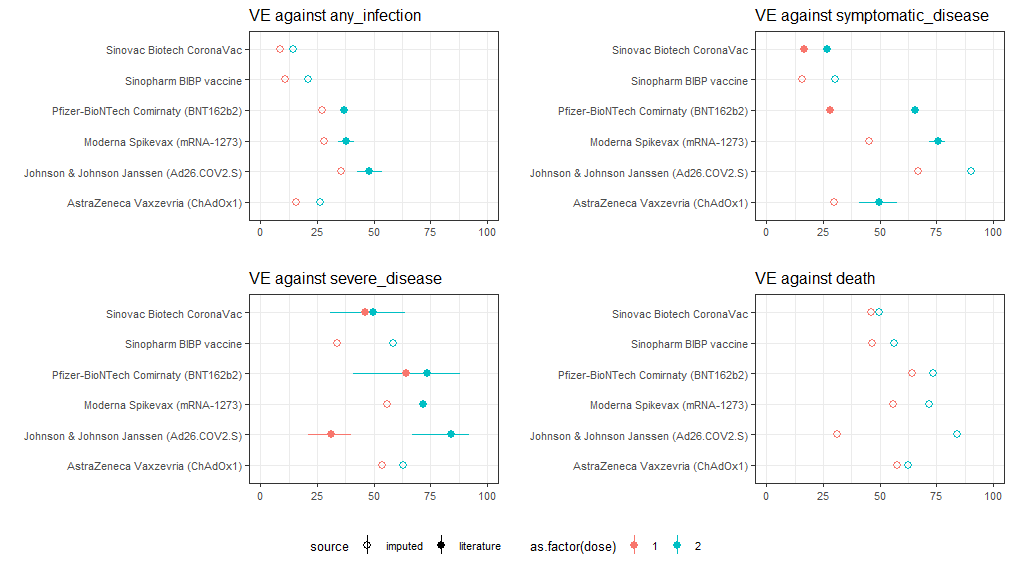


**Figure S2.2** Vaccine effectiveness estimates against the Omicron variant [13, 14, 24-29] as selected from [10]


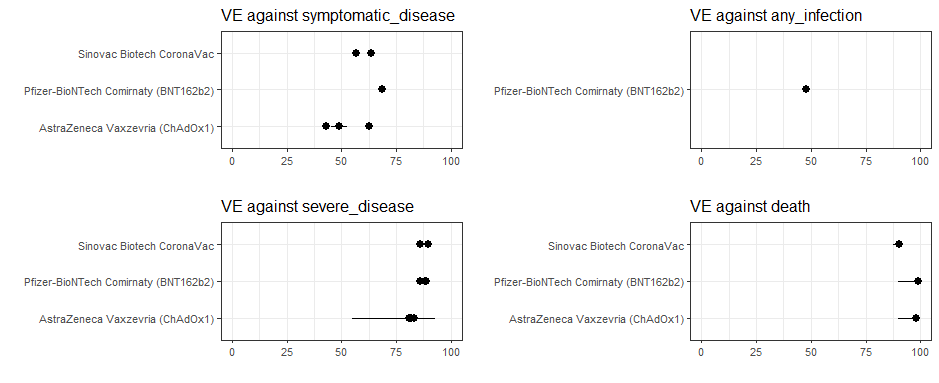


**Figure S2.3** Vaccine effectiveness estimates for heterologous combinations of a Pfizer booster dose [25, 26, 30-34] as selected from [10]

We estimated the speed of waning VE against infection by fitting an exponential distribution to data by Andrews et al. [34] (R^2^ 0.97 Moderna, 0.98 Pfizer and 0.8 AstraZeneca; Figure S2.4). Due to an absence of data, the waning of all viral (viral-vector and inactivated cell) vaccines was assumed to follow the waning of AstraZeneca. We also assumed that all doses of vaccination waned at this speed.


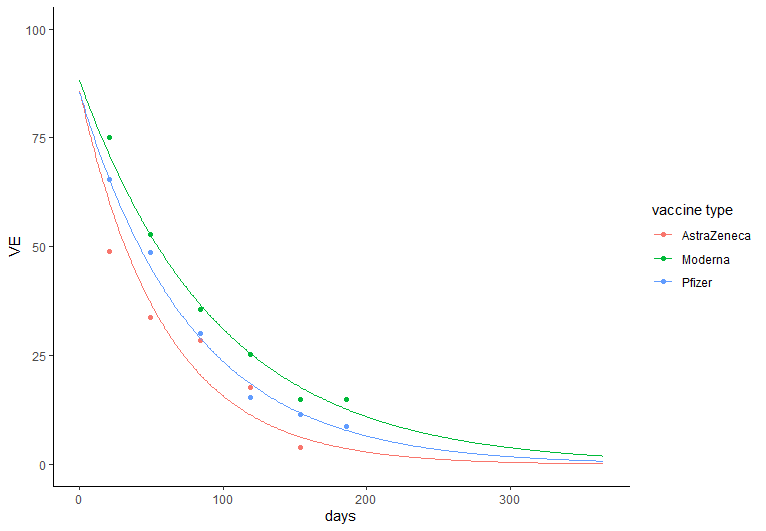


**Figure S2.4** Exponential fit to waning of vaccine effectiveness against acquisition, data from [34]

We modelled the waning of VE against severe outcomes as per Brazil data published in sequential studies by Cerqueira-Silva and colleagues [30, 35]. These studies estimated the effectiveness of two doses of Sinovac CoronaVac during the circulation of Delta, and two doses of Sinovac CoronaVac with a Pfizer booster during the circulation of Omicron. The study cohorts for these test-negative studies were large (14 and 2.5 million) as all individuals over the age of 18 who reported COVID-19 like symptoms and tested for COVID-19 were included using linked administrative data. There are limited data on single dose immunity, and on double dose immunity during the circulation of Omicron due to the ongoing rollout of vaccination programs in settings conducting large VE studies [10].

## **S2.3 Infection-derived immunity**

We modelled infection-derived immunity as clustered into pre-Omicron variants and Omicron variants [36]. We modelled the outbreak of an immune escape variant by including lower previous-to-new protection (e.g., Delta to Omicron) than like-to-like protection (e.g., Omicron BA.1/2 to Omicron BA.1/2) [37]. The speed at which the new variant outcompeted the previously circulating variant was estimated using the switch from Delta to Omicron in sequenced African samples reported to GISAID (Figure S2.5) [38].


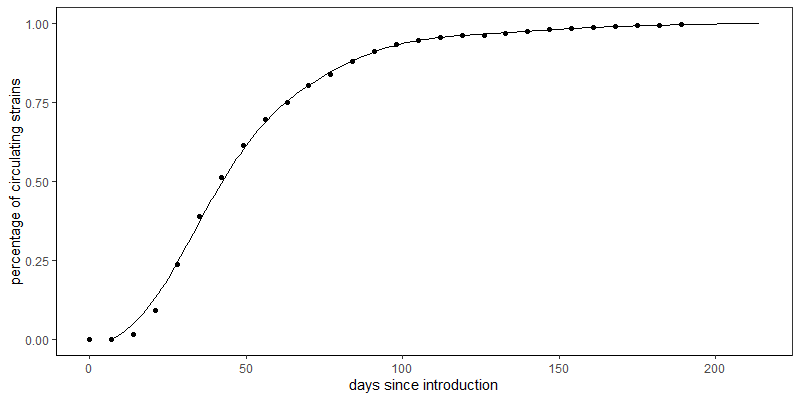


**Figure S2.5** Model estimate of the speed of introduction of a new immune escape as fit to Omicron in sequenced African samples from [38]

A review by Pilz and colleagues found a consensus that infection-derived protection against reinfection by the same variant appears robust over time, waning only moderately after 12 months [39]. We estimated ‘like-to-like’ protection by fitting an exponential curve to the results of national studies in Qatar estimating infection derived protection against Delta (Figure S2.6, R^2^=0.98) [36, 40, 41]. We estimated ‘previous-to-new’ protection from reinfection by a new immune-escape variant by fitting an exponential to the results of national studies conducted in Brazil and Qatar on protection of infection by a pre-Omicron variant against reinfection by Omicron (Figure S2.7, R^2^=0.83). Protection from prior infection against severe outcomes has appeared robust across variants, so we modelled infection-derived protection against severe outcomes as a constant 87.8% (95% CI 47.5-97.1) [40].

**Figure S2.6** Effectiveness of infection-derived immunity against Delta infection by time since infection

**Figure S2.7** Effectiveness of infection-derived immunity against Omicron infection by time since infection

Thus, we calculated infection-derived immunity ($\rho$) by considering protection against reinfection by the same variant (‘like-to-like’), and protection against reinfection by a new immune escape variant (‘previous-to-new’):

$$\rho_{old}= \sum_{days since recovery} \rho_{like-to-like}[days]$$

$$\rho_{new}= \sum_{days since recovery} \left( \% individuals recovered from previous strain* \rho_{prev-to-new}+\% individuals recovered from current strain* \rho_{like-to-like} \right)$$

$$\rho={(\% of circulating strain new variant*\rho}_{new})+(\% of circulating strain old variant* \rho_{old})$$

## **S2.4 Severe outcome projections**

We used estimates of infection severity in Sierra Leone to project from incidence to severe outcomes [42]. We incorporated an age-distribution into these estimates using their previous study (Figure S2.8) [43]. We adjusted these wild-type COVID-19 estimates to the increased severity of Delta and Omicron (Table S2.3) [44, 45].


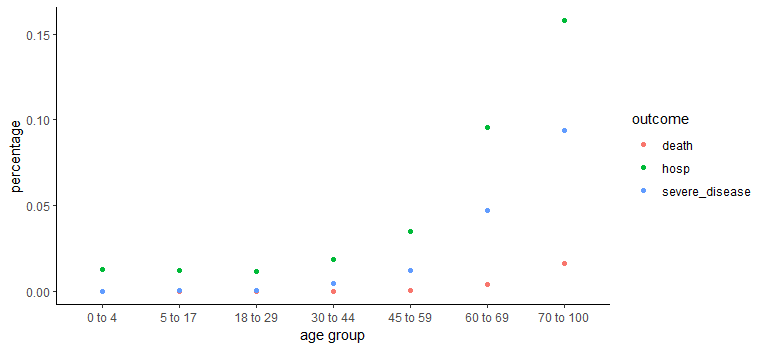
 **Figure S2.8** Age-distribution of severe outcomes

**Table S2.3** Comparison of infection severity estimates by circulating strain

| **Metric** | **(%)** |
| --- | --- |
| **Wild type** |  |
| Infection severity rate | 0.27 |
| Infection fatality rate | 0.034 |
| Infection acute-care bed hospitalisation rate | 0.125 |
| **Delta** |  |
| Infection severity rate | 0.90 |
| Infection fatality rate | 0.079 |
| Infection acute-care bed hospitalisation rate | 0.260 |
| **Omicron** |  |
| Infection severity rate | 0.52 |
| Infection fatality rate | 0.045 |
| Infection acute-care bed hospitalisation rate | 0.202 |

We calculated Years of Life Lost (YLL) using United Nations estimates of life expectancy in Sierra Leone during 2022 [46]. The average life expectancy per model age group was calculated using the age distribution of the study setting (Table S2.4). We did not include discounting of YLL. Adverse outcomes of pregnancy were not included in the calculation of YLL.

**Table S2.4** Calculation of years of life lost per modelled age group using United Nations estimates [46]

| **Age group** |  | **Number of remaining years of life expected** |
| --- | --- | --- |
| 0 to 4 |  | 62.7 |
| 5 to 9 |  | 60.1 |
| 10 to 17 |  | 54.2 |
| 18 to 29 |  | 45.4 |
| 30 to 44 |  | 33.7 |
| 45 to 59 |  | 21.4 |
| 60 to 69 |  | 12.3 |
| 70+ |  | 6.6 |

## **S2.5 Characterising high-risk groups**

**Adults with comorbidities**

We identified adults with comorbidities as those aged 30-59 with known comorbidities (1.2%) and all adults aged over 60 (4.7%), as per phase 1 & 2 of the Government of Sierra Leone’s COVAX plan [47], and using United Nations population estimates for 2022 [46]. There was limited literature to inform the increased risk of patients with co-morbidities. Meta-analysis to pool disparate studies has been difficult due to the use of different age-groups, and different measures - odds ratios, hazard ratios, risk ratios [48]. We used an estimate of 1.95 for the increased risk of severe COVID-19 based on WHO classification of severity as presented in multivariable logistic regression of Qatar data (95% CI 0.99, 3.82; p = 0.052) [49].

Access to primary care for the management of chronic conditions is limited in Sierra Leone. A national commission in 2020 reported that only 11% of health facilities offered services for diabetes and 20% for cardiovascular diseases, a majority being hospital-based services in urban settings [50]. A cross-sectional survey, also in 2020, estimated the cascade of care for diabetes and hypertension to be from 32.9% and 33.2% at diagnosis, to 19.0 and 14.7% accessing treatment, and 8.6% and 4.6% of cases achieving control of their condition [51]. The opportunistic vaccination of 10-20% of adults with comorbidities would increase the overall rate of vaccine rollout by 1.5%-3.0%. Given this small increase, we did not consider modelling a separate program for the opportunistic vaccination of adults with comorbidities who present to primary care.

**Pregnant Women**

We analysed the 2019 Demographic Health Survey of Sierra Leone to estimate the prevalence of women having given birth in the last year [52]. We utilised the R package *DHS.rates* to estimate age-specific fertility as per the official guide to DHS Statistics (Figure S2.9) [53, 54]. We then applied United Nations sex ratio estimates to derive population level estimates of pregnant women [46].


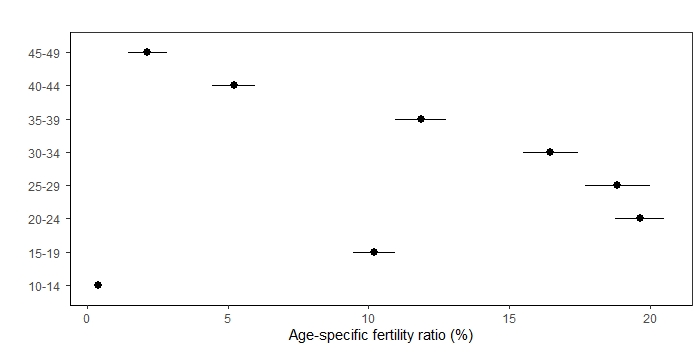


**Figure S2.9** Female age-specific fertility rates from analysis of Sierra Leone DHS 2019 [52]. Rates for the 10-14 age group are based on retrospective data and are presented without uncertainty.

We estimated that pregnant women were at 2.40 times higher risk of severe COVID-19 outcomes. This estimate comes from meta-analysis conducted by the UK Royal College of Obstetricians and Gynaecologists (RCOG) on the increased risk of ICU admissions for pregnant compared to non-pregnant women following COVID-19 (95% CI 2.25-2.57), including studies up to February 2022 [55]. We note that the RCOG acknowledge that most studies included in their meta-analysis were conducted when Alpha was the dominant strain, and that different variants bring variation in maternal health outcomes. While the RCOG meta-analysis includes studies from all income settings, there are a larger number of studies published in higher-income settings.

We also quantified the benefits of vaccination on pregnancy outcomes, considering the impact of COVID-19 infection on two adverse pregnancy outcomes: preterm delivery and stillbirth (Table S2.5). We used the results of a nationally representative mortality survey conducted in Sierra Leone between 2018-20 to estimate the baseline prevalence of neonatal deaths caused by these two adverse pregnancy outcomes [56]. We estimated the increased risk of these adverse outcomes leading to neonatal death through the odds ratios calculated by the RCOG meta-analysis. Due to an absence of data, we assumed equal vaccine-derived protection against adverse pregnancy outcomes as protection against severe disease in pregnant women. For simplicity, we assumed all births are singleton births and assumed no maternal age dependence on adverse pregnancy outcomes due to COVID-19 infection.

**Table S2.5** Calculation of adverse outcomes due to COVID-19 infection during pregnancy

| **Outcome** | **Baseline prevalence [56]** | **Increased risk [55]** |
| --- | --- | --- |
| Preterm delivery | 6.2 (95% CI 5.9-6.5) per 1000 livebirths | OR 1.47 (95% CI 1.44-1.51) |
| Stillbirth | 15.6 (95% CI 15.1-16.1) per 1000 livebirths | OR 1.80 (95% CI 1.63-1.99) |

# **S3. Model Fit**

We fitted the model to two reference points of seroprevalence: March 2021, and November 2021. We included pre-existing infection-derived immunity in the initial state of the model using a seroprevalence survey conducted in Sierra Leone in March 2021 [57]. This cross-sectional, nationally representative, and aged-stratified study estimated an overall seroprevalence of 2.6% (95% CI: 1.9% to 3.4%) after the first two waves of COVID-19 in Sierra Leone.

We introduced the Delta variant to the model in April to align with the reported Delta peak in Sierra Leone (Figure S3.1). At this point, the model switched from wild-type to Delta-specific estimates of key model parameters including the basic reproduction number, effectiveness of infection- and vaccine-derived immunity, length of the symptomatic period and latent period.

We ran the model until the 14^th^ of November 2021 and compared our model’s estimate of seroprevalence to a global meta-analysis estimate. This global meta-analysis used an ensemble model considering reported cases, hospital admissions, reported deaths, and seroprevalence surveys to estimate daily and cumulative COVID-19 infections [58]. Their model also included Global Burden of Disease estimates of the prevalence of comorbidities and the Healthcare Access and Quality (HAQ) Index. The global meta-analysis estimated that 40.5% (95% CI: 25.5-55.3%) of Sierra Leone was previously infected as of the 14^th of^ November 2021. In comparison, our model estimated that 57.9% of Sierra Leone had infection-derived immunity as of November 14^th^ 2021. Given that our model includes asymptomatic cases, and that there are known issues with the varied access to healthcare and diagnositic services in Sierra Leone, we believed this to be a reasonable fit.

We then introduced Omicron to the model to align with the Omicron peak of reported cases in Sierra Leone (Figure S3.1). Again, the model switched from Delta to Omicron-specific estimates of key model parameters. The introduction of Omicron as an immune-escape variant corresponded with a 1.59 increase in the effective reproduction number, which aligns with the World Health Organization estimate of 64% growth advantage of Omicron over Delta [59]. After the introduction of Omicron, the model reaches a steady state as anticipated by literature describing an endemic COVID-19 [60, 61]. Our model fit estimates that 43.5% of individuals have infection-derived immunity in August 2022 (see age distribution in Table S3.1). Figure S3.3 compares the age distribution of seroprevalence between March 2021, November 2021, and August 2022 (initial state of the model). Higher seroprevalence in adults relative to children early in the pandemic was likely a result of age-specific impacts of non-pharmaceutical interventions; in comparison, we expect higher seroprevalence in children later in the pandemic due to their higher contact leveles (Table S2.2) with the relaxing of non-pharmaceutical interventions and increased transmission potential of emerging variants. We provide sensitivity analysis for the influence of our model’s fit on our paper’s results by varying pre-existing infection derived immunity in S4.8.

**Table S3.1** Age-specific seroprevalence in initial state (August 2022) of the model after Omicron peak

| **Age group (years)** | **Seroprevalence (%)** |
| --- | --- |
| 0 to 4 | 42.7 |
| 5 to 9 | 51.1 |
| 10 to 17 | 51.7 |
| 18 to 29 | 42.4 |
| 30 to 44 | 39.1 |
| 45 to 59 | 35.3 |
| 60 to 69 | 32.5 |
| 70+ | 21.8 |

| **A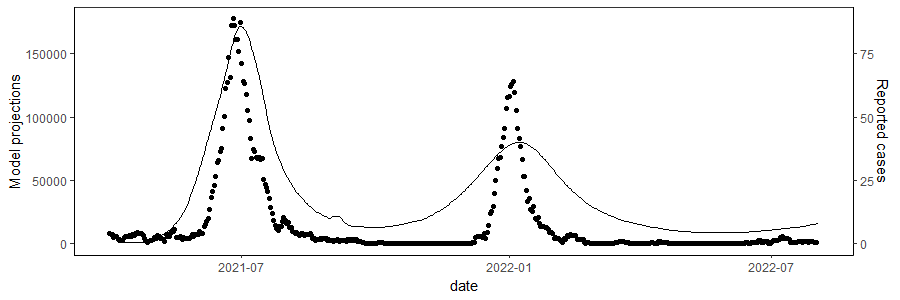** |
| --- |
| **B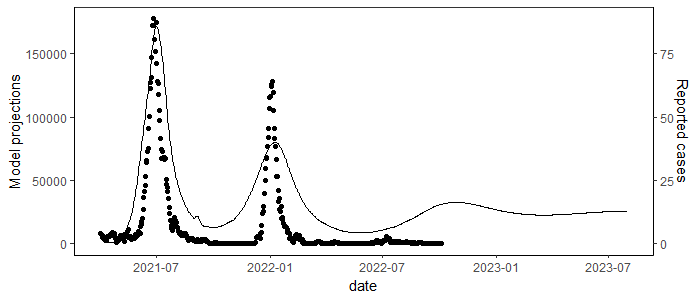** |

**Figure S3.1.** Comparison of model fit (line, left axis) to the daily reported number of cases (point, right axis). Panel A presents the model fit within the range of known data; Panel B extending the model by 52 weeks.


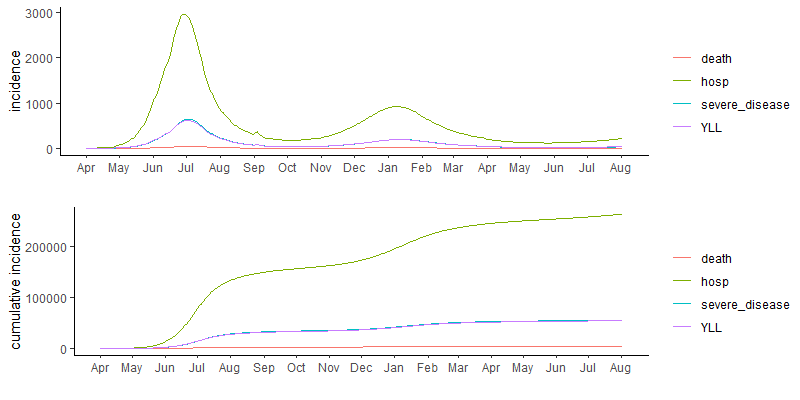


**Figure S3.2.** Health outcomes projected from model fit


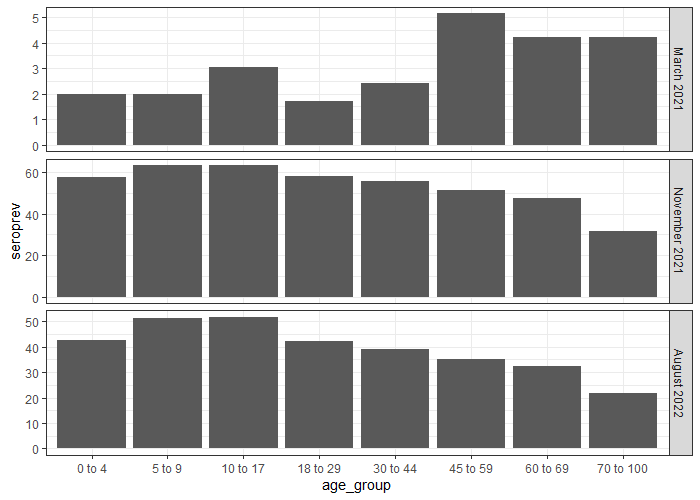


**Figure S3.3.** Comparison of the age distribution of seroprevalence across March 2021 (real data adapted), November 2021 (fitted to pop-level seroprevalence), and August 2022 (projected)

# **S4. Sensitivity Analysis**

## **S4.1 Outbreak of a new immune-escape variant**

We modelled the outbreak of a new immune-escape variant with equal inherent transmissibility to the previous Omicron sub variant. We assumed that the new variant outcompeted the existing strain at the speed which Omicron outcompeted Delta (see Figure S2.5). The outbreak of the new variant resulted in a temporary peak and returned to a steady state due to the high levels of infection-derived immunity in the population (Figure S4.1).


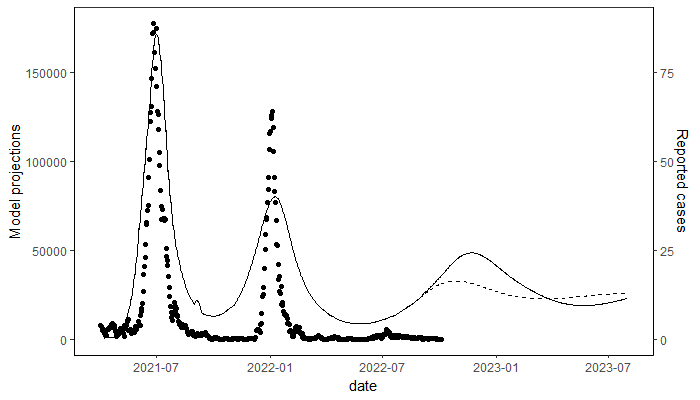


**Figure S4.1.** Outbreak of a new immune-escape variant (solid line) compared to no new variant (dashed line)

| **A**  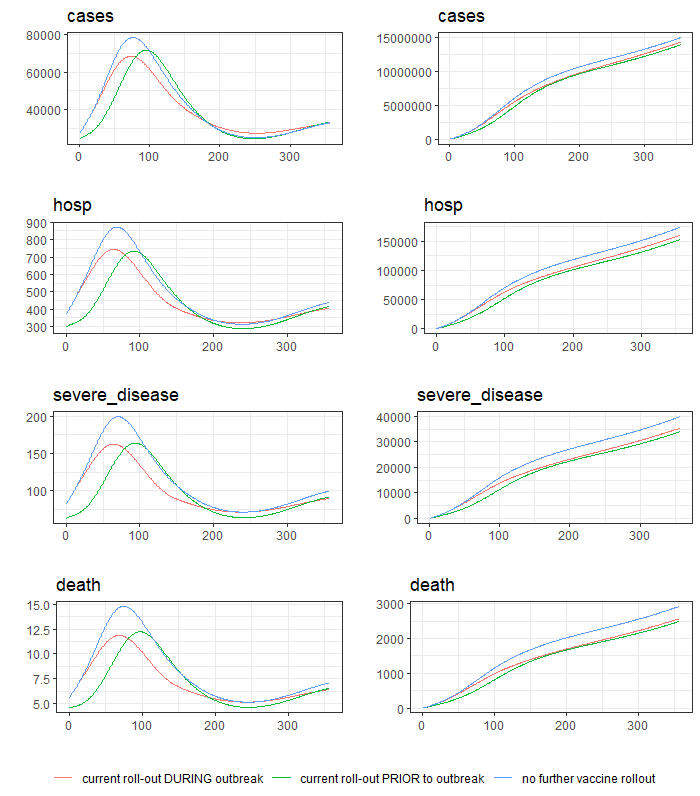 | **B**  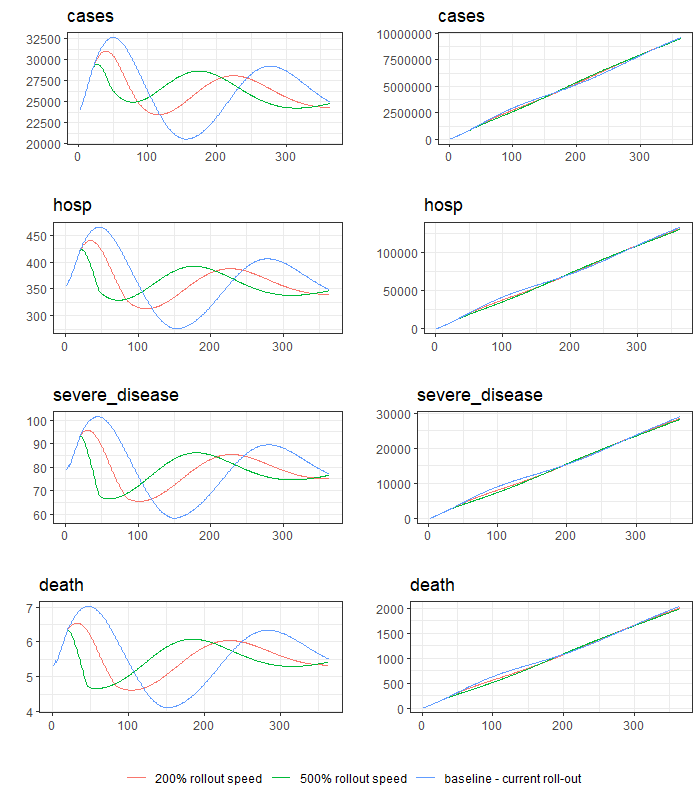 |
| --- | --- |

**Figure S4.2.** Comparison of different timings of vaccination relative to an outbreak (A), and varying speed of rollout during an outbreak (B). Absolute (left) and cumulative (right) values are presented. Baseline population coverage target of 51.6% with adult-eligibility only. Note the rollout ends at 170 days with the baseline speed of roll-out.

## **S4.2 Prioritisation of a double-dose vaccine**

We conducted sensitivity analysis for the prioritisation of a double-dose vaccine (Pfizer) in comparison to the use of a single-dose vaccine (Johnson & Johnson) in the main model. This sensitivity analysis assumed sufficient supply to reach the same level of coverage as the main model, i.e., double the vaccine supply with a double dose vaccine.

**Table S4.1** *Sensitivity analysis of main results using a double-dose vaccine.* Cumulative outcomes prevented by prioritisation strategies including children 5 to 17 years. Outcomes prevented calculated relative to current adult vaccination program with a population coverage target of 51.6%. Red/green shading indicates the increase/reduction of an outcome.

|  | **Children (0-4 years)** | | | | | **Children (5-17 years)** | | | | | **Adults (18+ years)** | | | | | **Overall** | | | | |
| --- | --- | --- | --- | --- | --- | --- | --- | --- | --- | --- | --- | --- | --- | --- | --- | --- | --- | --- | --- | --- |
|  | Cases | Severe disease | Hospitalisations | Deaths | YLL | Cases | Severe disease | Hospitalisations | Deaths | YLL | Cases | Severe disease | Hospitalisations | Deaths | YLL | Cases | Severe disease | Hospitalisations | Deaths | YLL |
| **Expanding to children concurrently with the adult rollout** | | | | | | | | | | | | | | | | | | | | |
| 51.6% | 28326 | 3 | 331 | 1 | 39 | -32874 | -62 | -3698 | -6 | -328 | 231037 | 3504 | 11182 | 245 | 2896 | 226489 | 3445 | 7815 | 239 | 2606 |
|  | 2.3% | 2.2% | 2.2% | 2.2% | 2.2% | -0.9% | -9.1% | -9.1% | -9.1% | -9.1% | 5.6% | 19.2% | 19.0% | 19.3% | 19.3% | 2.5% | 18.1% | 6.8% | 17.5% | 12.8% |
| 60% | 14266 | 2 | 167 | 0 | 19 | -96095 | -85 | -5097 | -9 | -452 | 165094 | 2924 | 9378 | 202 | 2408 | 83264 | 2840 | 4449 | 194 | 1975 |
|  | 1.1% | 1.1% | 1.1% | 1.1% | 1.1% | -2.6% | -12.5% | -12.5% | -12.5% | -12.5% | 4.0% | 16.0% | 15.9% | 16.0% | 16.1% | 0.9% | 14.9% | 3.9% | 14.2% | 9.7% |
| 70% | -6341 | -1 | -80 | 0 | -9 | -189826 | -108 | -6465 | -11 | -574 | 75795 | 2320 | 7475 | 158 | 1903 | -120372 | 2211 | 930 | 147 | 1320 |
|  | -0.5% | -0.5% | -0.5% | -0.5% | -0.5% | -5.2% | -15.9% | -15.9% | -15.9% | -15.8% | 1.8% | 12.7% | 12.7% | 12.5% | 12.7% | -1.3% | 11.6% | 0.8% | 10.8% | 6.5% |
| 75.5% | -9883 | -1 | -123 | 0 | -14 | -215050 | -113 | -6789 | -11 | -602 | 57226 | 2197 | 7084 | 150 | 1801 | -167706 | 2082 | 171 | 138 | 1184 |
|  | -0.8% | -0.8% | -0.8% | -0.8% | -0.8% | -5.9% | -16.7% | -16.7% | -16.6% | -16.6% | 1.4% | 12.1% | 12.0% | 11.8% | 12.0% | -1.9% | 10.9% | 0.1% | 10.1% | 5.8% |
| **Expanding to children after adult rollout** | | | | | | | | | | | | | | | | | | | | |
| 60% | 6183 | 1 | 74 | 0 | 9 | 12374 | 2 | 131 | 0 | 12 | 34732 | 291 | 927 | 20 | 240 | 53289 | 294 | 1132 | 21 | 261 |
|  | 0.5% | 0.5% | 0.5% | 0.5% | 0.5% | 0.3% | 0.3% | 0.3% | 0.3% | 0.3% | 0.8% | 1.6% | 1.6% | 1.6% | 1.6% | 0.6% | 1.5% | 1.0% | 1.5% | 1.3% |
| 70% | -12500 | -2 | -151 | 0 | -18 | -80292 | -22 | -1339 | -2 | -119 | -42792 | -185 | -573 | -14 | -158 | -135584 | -209 | -2063 | -17 | -294 |
|  | -1.0% | -1.0% | -1.0% | -1.0% | -1.0% | -2.2% | -3.3% | -3.3% | -3.3% | -3.3% | -1.0% | -1.0% | -1.0% | -1.1% | -1.1% | -1.5% | -1.1% | -1.8% | -1.2% | -1.4% |
| 75.5% | -15211 | -2 | -184 | 0 | -21 | -110045 | -30 | -1778 | -3 | -158 | -51858 | -225 | -696 | -17 | -192 | -177114 | -256 | -2658 | -21 | -371 |
|  | -1.2% | -1.2% | -1.2% | -1.2% | -1.2% | -3.0% | -4.4% | -4.4% | -4.4% | -4.4% | -1.3% | -1.2% | -1.2% | -1.4% | -1.3% | -2.0% | -1.3% | -2.3% | -1.5% | -1.8% |

**Table S4.2** *Sensitivity analysis of main results using a double-dose vaccine.* Cumulative outcomes prevented by risk-specific prioritisation strategies. Outcomes prevented calculated relative to current adult vaccination program with a population coverage target of 51.6% and uniform eligibility. Red/green shading indicates the increase/reduction of an outcome.

|  | Incidence | Incidence of severe disease | Hospitalisations | Deaths | YLL | Neonatal deaths |
| --- | --- | --- | --- | --- | --- | --- |
| Pregnant women | | | | | | |
| **Prioritising within existing rollout capacity** | | | | | | |
| 25% primary dose only | -4,878 (-0.1%) | 50 (0.3%) | -67 (-0.1%) | 7 (0.5%) | 62 (0.3%) | -185 (-6.3%) |
| 50% primary dose only | -155 (0.0%) | 25 (0.1%) | -149 (-0.1%) | 6 (0.4%) | 46 (0.2%) | -214 (-7.3%) |
| 75% primary dose only | -28 (0.0%) | 26 (0.1%) | -148 (-0.1%) | 6 (0.4%) | 47 (0.2%) | -217 (-7.4%) |
| 50% primary and booster provision | -50,855 (-0.6%) | -63 (-0.3%) | -1,498 (-1.3%) | 14 (1.0%) | 26 (0.1%) | -1,054 (-36.0%) |
| **Additional rollout capacity (antenatal visits)** | | | | | | |
| Additional primary doses | 20,685 (0.2%) | -115 (-0.6%) | -455 (-0.4%) | -5 (-0.3%) | -63 (-0.3%) | -204 (-7.0%) |
| Additional primary and booster doses | 4,632 (0.1%) | -414 (-2.2%) | -2,314 (-2.0%) | -11 (-0.8%) | -239 (-1.2%) | -1,112 (-38.0%) |
| Expanding eligibility to high-risk children | 16,264 (0.2%) | -123 (-0.6%) | -965 (-0.8%) | -6 (-0.4%) | -103 (-0.5%) | -500 (-17.1%) |
| Adults with comorbidities | | | | | | |
| **Prioritising within existing rollout capacity** | | | | | | |
| 25% primary dose only | -6,208 (-0.1%) | -409 (-2.1%) | -741 (-0.6%) | -48 (-3.5%) | -434 (-2.1%) | NA |
| 50% primary dose only | -11,616 (-0.1%) | -665 (-3.5%) | -1,170 (-1.0%) | -80 (-5.8%) | -713 (-3.5%) | NA |
| 75% primary dose only | -1,195 (0.0%) | -815 (-4.3%) | -1,451 (-1.3%) | -95 (-6.9%) | -854 (-4.2%) | NA |
| 50% primary and booster provision | -99,426 (-1.1%) | -3,447 (-18.1%) | -7,153 (-6.2%) | -459 (-33.6%) | -4,293 (-21.1%) | NA |
| **Additional rollout capacity** | | | | | | |
| Additional primary doses | 50,763 (0.6%) | -910 (-4.8%) | -1,799 (-1.6%) | -91 (-6.7%) | -860 (-4.2%) | NA |
| Additional primary and booster doses | 13,409 (0.1%) | -4,986 (-26.2%) | -10,482 (-9.1%) | -622 (-45.6%) | -5,894 (-28.9%) | NA |

## **S4.3 Prioritisation of a larger number of doses**

The main paper presents analysis for the prioritisation of doses from October 2022 onwards, with 1,381,537 doses (17.5% of the population) left to allocate. Here, we present additional analysis to quantify the benefit of prioritising high-risk groups for primary doses from the start of 2022, as proposed by the Government of Sierra Leone in their original COVAX plan [47]. We also present analysis of the prioritisation of high-risk groups for hypothetical booster doses in 2023. This additional analysis assumed that a booster dose would be eligible to all adults aged over 18 who have received their primary schedule.

**Table S4.3** *Sensitivity analysis for the prioritisation of high-risk groups for primary doses in 2022, or booster doses in 2023.* Cumulative outcomes prevented by risk-specific prioritisation strategies. Outcomes prevented calculated relative to current adult vaccination program with a population coverage target of 51.6% and uniform eligibility. Red/green shading indicates the increase/reduction of an outcome.

|  | Incidence | Incidence of severe disease | Hospitalisations | Deaths | YLL | Neonatal deaths |
| --- | --- | --- | --- | --- | --- | --- |
| Pregnant women | | | | | | |
| **Prioritising primary doses in 2022** | | | | | | |
| 25% rollout capacity | 2,622 (0.0%) | 34 (0.1%) | -17 (0.0%) | 5 (0.3%) | 44 (0.2%) | -108 (-3.2%) |
| 50% rollout capacity | 5,311 (0.1%) | 58 (0.2%) | 4 (0.0%) | 8 (0.5%) | 72 (0.3%) | -154 (-4.6%) |
| 75% rollout capacity | 7,482 (0.1%) | 73 (0.3%) | 24 (0.0%) | 9 (0.5%) | 87 (0.4%) | -175 (-5.2%) |
| **Prioritising booster doses in 2023** | | | | | | |
| 25% rollout capacity | 1,257 (0.0%) | 75 (0.3%) | -218 (-0.1%) | 13 (0.8%) | 114 (0.5%) | -434 (-12.1%) |
| 50% rollout capacity | 2,711 (0.0%) | 91 (0.4%) | -227 (-0.2%) | 15 (0.9%) | 134 (0.5%) | -489 (-13.7%) |
| 75% rollout capacity | 4,194 (0.0%) | 99 (0.4%) | -219 (-0.2%) | 16 (1.0%) | 143 (0.6%) | -508 (-14.2%) |
| Adults with comorbidities | | | | | | |
| **Prioritising primary doses in 2022** | | | | | | |
| 25% rollout capacity | -3,478 (0.0%) | -211 (-0.9%) | -384 (-0.3%) | -25 (-1.4%) | -223 (-0.9%) | NA |
| 50% rollout capacity | -908 (0.0%) | -436 (-1.8%) | -766 (-0.6%) | -53 (-3.0%) | -465 (-1.9%) | NA |
| 75% rollout capacity | 3,742 (0.0%) | -588 (-2.5%) | -1,011 (-0.8%) | -72 (-4.1%) | -631 (-2.6%) | NA |
| **Prioritising booster doses in 2023** | | | | | | |
| 25% rollout capacity | -5,283 (0.0%) | -1,067 (-4.7%) | -1,878 (-1.3%) | -124 (-7.6%) | -1,117 (-4.5%) | NA |
| 50% rollout capacity | -6,975 (-0.1%) | -1,593 (-7.0%) | -2,785 (-1.9%) | -186 (-11.4%) | -1,670 (-6.7%) | NA |
| 75% rollout capacity | -3,971 (0.0%) | -1,763 (-7.8%) | -3,055 (-2.1%) | -206 (-12.7%) | -1,849 (-7.4%) | NA |

## **S4.4 Increased vaccine hesitancy in pregnant women**

We conducted sensitivity analysis for lower vaccine acceptance in pregnant women – 70% compared to baseline 88%. This sensitivity analysis was inspired by a survey of 16 countries conducted in late 2020 [62]. The survey reported lower vaccine acceptance in pregnant women (52.0%, range 28.8%-84.4%) compared to non-pregnant women (73.4%, range 48.6%-93.1%). Notably, COVID-19 vaccine acceptance was much higher among pregnant women in low- and middle-income settings (>85% in India and Mexico) compared to high-income settings (<45% in US, Australia, and Russia) [62]. A key concern of vaccine hesitant women (48.8%) was the lack of data on safety and efficacy of COVID-19 vaccines during pregnancy [62]. Pregnant women were not included in the initial randomised controlled trials of COVID-19 vaccines due to safety concerns [63, 64]. We expect vaccine acceptance to have risen over time as data on the safety and efficacy of COVID-19 vaccines among pregnant women has emerged [65]. As such, this sensitivity analysis is a pessimistic estimate of vaccine acceptance in Sierra Leone. Nevertheless, sensitivity analysis supports similar effects for the risk-specific prioritisation of pregnant women (Table S4.4 compared to Table 3 in main paper). The benefit of prioritising pregnant women is simply muted with a reduced uptake of vaccine.

**Table S4.4** *Sensitivity analysis for the increased vaccine hesitancy in pregnant women.* Cumulative outcomes for the prioritisation of pregnant women presented. Outcomes prevented calculated relative to current vaccination program with a population coverage target of 51.6% and uniform eligibility.

|  | Incidence | Incidence of severe disease | Hospitalisations | Deaths | YLL | Neonatal deaths |
| --- | --- | --- | --- | --- | --- | --- |
| **Prioritising within existing rollout capacity** | | | | | | |
| 25% primary dose only | -127 (0.0%) | 0  (0.0%) | -1  (0.0%) | 0 (0.0%) | 0 (0.0%) | 0  (0.0%) |
| 50% primary dose only | -36  (0.0%) | 0  (0.0%) | 0  (0.0%) | 0 (0.0%) | 0 (0.0%) | 0  (0.0%) |
| 75% primary dose only | -8  (0.0%) | 0  (0.0%) | 0  (0.0%) | 0 (0.0%) | 0 (0.0%) | 0  (0.0%) |
| 50% primary and booster provision | -19,259  (-0.2%) | -240  (-1.1%) | -1,687  (-1.4%) | 0  (0.0%) | -87  (-0.4%) | -956  (-27.9%) |
| **Additional rollout capacity (antenatal visits)** | | | | | | |
| Additional primary doses | -1,325 (0.0%) | -11  (-0.1%) | -45  (0.0%) | -1  (-0.1%) | -10 (0.0%) | 0  (0.0%) |
| Additional primary and booster doses | -26,146  (-0.3%) | -339  (-1.6%) | -2,052  (-1.7%) | -8  (-0.5%) | -175  (-0.8%) | -950  (-27.7%) |
| Expanding eligibility to high-risk children | -4,084 (0.0%) | -17  (-0.1%) | -262  (-0.2%) | -1  (-0.1%) | -27  (-0.1%) | -119  (-3.5%) |

## **S4.5 Reduced vaccine effectiveness in older adults**

We examined the impact of age-, dose- and risk-prioritisation strategies if older adults had lower protection from vaccination against severe outcomes. Studies have suggested that older adults may experience a lower protection from vaccination and a faster waning of protection against severe outcomes [26, 30, 35, 66]. There have been no statistically significant age dependent differences reported in VE against infection or symptomatic disease [26, 67].

For this sensitivity analysis, we used the ratio of VE between age groups, and age-stratified estimates of waning VE against severe outcomes reported by Cerqueira-Silva et al. [30, 35]. This ratio and speed of waning were applied to the point estimates presented in S2.2. The data suggested that immunity derived from a primary schedule waned faster than immunity from a booster dose (Figure S4.3).


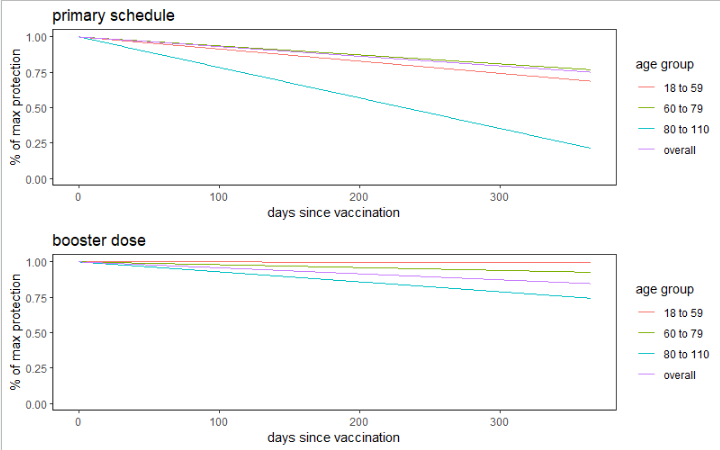
**Figure S4.3** Speed of waning immunity against severe outcomes (adapted from [30, 35])

**Table S4.5** *Sensitivity analysis of main results assuming lower vaccine-derived protection against severe outcomes in older adults.* Cumulative outcomes prevented by prioritisation strategies including children 5 to 17 years. Outcomes prevented calculated relative to current adult vaccination program with a population coverage target of 51.6%. Red/green shading indicates the increase/reduction of an outcome.

|  | **Children (0-4 years)** | | | | | **Children (5-17 years)** | | | | | **Adults (18+ years)** | | | | | **Overall** | | | | |
| --- | --- | --- | --- | --- | --- | --- | --- | --- | --- | --- | --- | --- | --- | --- | --- | --- | --- | --- | --- | --- |
|  | Cases | Severe disease | Hospitalisations | Deaths | YLL | Cases | Severe disease | Hospitalisations | Deaths | YLL | Cases | Severe disease | Hospitalisations | Deaths | YLL | Cases | Severe disease | Hospitalisations | Deaths | YLL |
| **Expanding to children concurrently with the adult rollout** | | | | | | | | | | | | | | | | | | | | |
| 51.6% | 3079 | 0 | 39 | 0 | 4 | -78868 | -67 | -4020 | -7 | -357 | 61445 | 722 | 3301 | 18 | 441 | -14343 | 655 | -680 | 11 | 89 |
|  | 0.2% | 0.2% | 0.2% | 0.2% | 0.2% | -2.1% | -9.8% | -9.8% | -9.8% | -9.8% | 1.4% | 3.6% | 5.1% | 1.2% | 2.6% | -0.2% | 3.1% | -0.6% | 0.7% | 0.4% |
| 60% | -4198 | 0 | -39 | 0 | -5 | -131599 | -91 | -5488 | -9 | -487 | 15941 | 395 | 2094 | 0 | 196 | -119856 | 304 | -3432 | -10 | -296 |
|  | -0.3% | -0.3% | -0.3% | -0.3% | -0.3% | -3.5% | -13.3% | -13.3% | -13.3% | -13.3% | 0.4% | 2.0% | 3.2% | 0.0% | 1.2% | -1.3% | 1.4% | -2.8% | -0.6% | -1.3% |
| 70% | -33018 | -4 | -381 | -1 | -44 | -258844 | -123 | -7384 | -12 | -656 | -109179 | -301 | -258 | -46 | -362 | -401040 | -428 | -8023 | -59 | -1062 |
|  | -2.6% | -2.5% | -2.5% | -2.5% | -2.5% | -7.0% | -17.9% | -17.9% | -17.9% | -17.9% | -2.5% | -1.5% | -0.4% | -3.2% | -2.2% | -4.3% | -2.0% | -6.6% | -3.9% | -4.8% |
| 75.5% | -46812 | -6 | -548 | -1 | -64 | -329889 | -137 | -8204 | -14 | -728 | -168703 | -602 | -1273 | -66 | -605 | -545404 | -744 | -10025 | -81 | -1397 |
|  | -3.7% | -3.5% | -3.5% | -3.5% | -3.5% | -8.9% | -19.9% | -19.9% | -19.9% | -19.9% | -3.9% | -3.0% | -2.0% | -4.6% | -3.6% | -5.9% | -3.5% | -8.3% | -5.3% | -6.3% |
| **Expanding to children after adult rollout** | | | | | | | | | | | | | | | | | | | | |
| 60% | -6465 | -1 | -66 | 0 | -8 | -91966 | -54 | -3267 | -6 | -290 | -23829 | -149 | -384 | -14 | -141 | -122260 | -204 | -3717 | -20 | -439 |
|  | -0.5% | -0.4% | -0.4% | -0.4% | -0.4% | -2.5% | -7.9% | -7.9% | -7.9% | -7.9% | -0.6% | -0.7% | -0.6% | -1.0% | -0.8% | -1.3% | -1.0% | -3.1% | -1.3% | -2.0% |
| 70% | -33170 | -4 | -384 | -1 | -45 | -244179 | -96 | -5754 | -10 | -511 | -117758 | -652 | -1896 | -54 | -576 | -395107 | -752 | -8034 | -65 | -1131 |
|  | -2.6% | -2.5% | -2.5% | -2.5% | -2.5% | -6.6% | -14.0% | -14.0% | -14.0% | -14.0% | -2.7% | -3.2% | -2.9% | -3.8% | -3.4% | -4.3% | -3.6% | -6.6% | -4.2% | -5.1% |
| 75.5% | -45825 | -6 | -537 | -1 | -62 | -329830 | -113 | -6787 | -11 | -602 | -161193 | -879 | -2592 | -72 | -772 | -536848 | -998 | -9917 | -85 | -1436 |
|  | -3.6% | -3.5% | -3.5% | -3.5% | -3.5% | -8.9% | -16.5% | -16.5% | -16.5% | -16.5% | -3.8% | -4.4% | -4.0% | -5.0% | -4.6% | -5.8% | -4.7% | -8.2% | -5.5% | -6.5% |

**Table S4.6** *Sensitivity analysis of main results assuming lower vaccine-derived protection against severe outcomes in older adults.* Cumulative outcomes prevented by risk-specific prioritisation strategies. Outcomes prevented calculated relative to current adult vaccination program with a population coverage target of 51.6% and uniform eligibility. Red/green shading indicates the increase/reduction of an outcome.

|  | Incidence | Incidence of severe disease | Hospitalisations | Deaths | YLL | Neonatal deaths |
| --- | --- | --- | --- | --- | --- | --- |
| Pregnant women | | | | | | |
| **Prioritising within existing rollout capacity** | | | | | | |
| 25% primary dose only | 779 (0.0%) | 5 (0.0%) | -13 (0.0%) | 1 (0.1%) | 8 (0.0%) | -31 (-1.0%) |
| 50% primary dose only | 1,142 (0.0%) | 7 (0.0%) | -10 (0.0%) | 1 (0.1%) | 9 (0.0%) | -33 (-1.1%) |
| 75% primary dose only | 1,278 (0.0%) | 7 (0.0%) | -8 (0.0%) | 1 (0.1%) | 10 (0.0%) | -34 (-1.1%) |
| 50% primary and booster provision | -24,466 (-0.3%) | -346 (-1.6%) | -2,453 (-2.0%) | -1 (0.0%) | -124 (-0.6%) | -1,396 (-44.4%) |
| **Additional rollout capacity (antenatal visits)** | | | | | | |
| Additional primary doses | -2,591 (0.0%) | -35 (-0.2%) | -171 (-0.1%) | -2 (-0.1%) | -28 (-0.1%) | -31 (-1.0%) |
| Additional primary and booster doses | -31,022 (-0.3%) | -463 (-2.2%) | -2,872 (-2.4%) | -9 (-0.6%) | -228 (-1.0%) | -1,375 (-43.8%) |
| Expanding eligibility to high-risk children | -5,754 (-0.1%) | -42 (-0.2%) | -443 (-0.4%) | -2 (-0.2%) | -49 (-0.2%) | -184 (-5.9%) |
| Adults with comorbidities | | | | | | |
| **Prioritising within existing rollout capacity** | | | | | | |
| 25% primary dose only | 1,339 (0.0%) | -64 (-0.3%) | -98 (-0.1%) | -7 (-0.5%) | -67 (-0.3%) | NA |
| 50% primary dose only | 3,106 (0.0%) | -83 (-0.4%) | -113 (-0.1%) | -10 (-0.6%) | -86 (-0.4%) | NA |
| 75% primary dose only | 4,007 (0.0%) | -84 (-0.4%) | -107 (-0.1%) | -10 (-0.6%) | -88 (-0.4%) | NA |
| 50% primary and booster provision | -46,484 (-0.5%) | -5,193 (-24.5%) | -10,465 (-8.6%) | -562 (-36.3%) | -5,214 (-23.4%) | NA |
| **Additional rollout capacity** | | | | | | |
| Additional primary doses | -5,738 (-0.1%) | -138 (-0.7%) | -436 (-0.4%) | -11 (-0.7%) | -127 (-0.6%) | NA |
| Additional primary and booster doses | -55,330 (-0.6%) | -5,737 (-27.1%) | -11,997 (-9.9%) | -611 (-39.5%) | -5,722 (-25.6%) | NA |

## **S4.6 Reduced vaccine effectiveness in older adults and adults with comorbidities**

We also examined the impact of age-, dose- and risk-prioritisation strategies if adults with comorbidities had lower protection from vaccination against severe outcomes. Recent studies report a statistically significant trend of decreased VE against severe outcomes with increased number of comorbidities [66, 68]. There has been no observed differences in VE against symptomatic disease [69], or in the speed of waning of vaccine-derived immunity in adults with comorbidities [27, 66].

To conduct this sensitivity analysis, we carried across estimates from our previous sensitivity analysis for older adults (S4.4) and applied a 10% reduction to VE against severe outcomes in adults with comorbidities aged 30-59 (average across [66, 68]). Recall that we identified our high-risk group of adults as adults aged 30-59 with known comorbidities and all adults aged over 60, as per the Government of Sierra Leone’s COVAX plan [47].

**Table S4.7** *Sensitivity analysis of main results assuming lower vaccine-derived protection against severe outcomes in older adults and adults with comorbidities.* Cumulative outcomes prevented by risk-specific prioritisation strategies. Outcomes prevented calculated relative to current adult vaccination program with a population coverage target of 51.6% and uniform eligibility. Red/green shading indicates the increase/reduction of an outcome.

|  | Incidence | Incidence of severe disease | Hospitalisations | Deaths | YLL |
| --- | --- | --- | --- | --- | --- |
| Adults with comorbidities | | | | | |
| **Prioritising within existing rollout capacity** | | | | | |
| 25% primary dose only | 1,339  (0.0%) | -63  (-0.3%) | -93  (-0.1%) | -7  (-0.5%) | -66  (-0.3%) |
| 50% primary dose only | 3,106  (0.0%) | -81  (-0.4%) | -105  (-0.1%) | -9  (-0.6%) | -85  (-0.4%) |
| 75% primary dose only | 4,007  (0.0%) | -82  (-0.4%) | -98  (-0.1%) | -10  (-0.6%) | -86  (-0.4%) |
| 50% primary and booster provision | -46,484  (-0.5%) | -5,133  (-24.1%) | -10,261  (-8.4%) | -561  (-36.2%) | -5,180  (-23.2%) |
| **Additional rollout capacity** | | | | | |
| Additional primary doses | -5,738  (-0.1%) | -136  (-0.6%) | -428  (-0.4%) | -11  (-0.7%) | -126  (-0.6%) |
| Additional primary and booster doses | -55,330  (-0.6%) | -5,668  (-26.7%) | -11,765  (-9.6%) | -609  (-39.3%) | -5,683  (-25.4%) |

## **S4.7 Increased or decreased risk of high-risk groups**

We conducted sensitivity analysis for the effects of prioritising pregnant women or adults with comorbidities if their risk of developing severe outcomes was equal to, 1.5 times, or 3 times that of the general population. See Table 3 in the main paper for results using baseline assumptions of 1.95 times increased risk for adults with comorbidities and 2.4 times increased risk for pregnant women (see S2.5).

The prioritisation of adults with comorbidities was highly beneficial regardless of their relative risk because of the older age demographic of the group, and the increased risk of severe COVID-19 outcomes with age. The prioritisation of pregnant women had minimal effect when they had equal risk compared to the general population. The benefit of prioritising pregnant women improved as their relative risk to general population increased. The opportunistic vaccination of both groups was beneficial in all scenarios.

**Table S4.8** Sensitivity analysis for prioritisation of high-risk groups where the individuals have **no increased risk** compared to the general population. Severe outcomes applicable to the general population presented only.

|  | Incidence of severe disease | Hospitalisations | Deaths | YLL |
| --- | --- | --- | --- | --- |
| Pregnant women | | | | |
| **Prioritising within existing rollout capacity** | | | | |
| 25% primary dose only | 11 (0.1%) | 23 (0.0%) | 1 (0.1%) | 11 (0.0%) |
| 50% primary dose only | 13 (0.1%) | 28 (0.0%) | 1 (0.1%) | 12 (0.1%) |
| 75% primary dose only | 13 (0.1%) | 30 (0.0%) | 1 (0.1%) | 13 (0.1%) |
| 50% primary and booster provision | -86 (-0.4%) | -796 (-0.7%) | 3 (0.2%) | -7 (0.0%) |
| **Additional rollout capacity (opportunistic vaccination during antenatal visits)** | | | | |
| Additional primary doses | -31 (-0.1%) | -141 (-0.1%) | -2 (-0.1%) | -26 (-0.1%) |
| Additional primary and booster doses | -211 (-1.0%) | -1,257 (-1.0%) | -6 (-0.4%) | -117 (-0.5%) |
| Expanding eligibility to high-risk children | -35 (-0.2%) | -257 (-0.2%) | -2 (-0.1%) | -36 (-0.2%) |
| Adults with comorbidities | | | | |
| **Prioritising within existing rollout capacity** | | | | |
| 25% primary dose only | -62 (-0.3%) | -85 (-0.1%) | -8 (-0.5%) | -69 (-0.3%) |
| 50% primary dose only | -81 (-0.4%) | -95 (-0.1%) | -11 (-0.7%) | -90 (-0.4%) |
| 75% primary dose only | -83 (-0.4%) | -88 (-0.1%) | -11 (-0.7%) | -93 (-0.4%) |
| 50% primary and booster provision | -4,494 (-21.2%) | -8,569 (-7.0%) | -512 (-33.8%) | -4,657 (-21.0%) |
| **Additional rollout capacity (opportunistic vaccination during regular care visits)** | | | | |
| Additional primary doses | -138 (-0.6%) | -422 (-0.3%) | -12 (-0.8%) | -132 (-0.6%) |
| Additional primary and booster doses | -4,962 (-23.4%) | -9,899 (-8.1%) | -555 (-36.6%) | -5,102 (-23.0%) |

**Table S4.9** Sensitivity analysis for prioritisation of high-risk groups where the individuals have **1.5 times the risk** of the general population. Severe outcomes applicable to the general population presented only.

|  | Incidence of severe disease | Hospitalisations | Deaths | YLL |
| --- | --- | --- | --- | --- |
| Pregnant women | | | | |
| **Prioritising within existing rollout capacity** | | | | |
| 25% primary dose only | 9 (0.0%) | 10 (0.0%) | 1 (0.1%) | 10 (0.0%) |
| 50% primary dose only | 11 (0.0%) | 15 (0.0%) | 1 (0.1%) | 12 (0.1%) |
| 75% primary dose only | 11 (0.1%) | 17 (0.0%) | 1 (0.1%) | 12 (0.1%) |
| 50% primary and booster provision | -164 (-0.8%) | -1,298 (-1.1%) | 2 (0.1%) | -42 (-0.2%) |
| **Additional rollout capacity (opportunistic vaccination during antenatal visits)** | | | | |
| Additional primary doses | -33 (-0.2%) | -151 (-0.1%) | -2 (-0.1%) | -27 (-0.1%) |
| Additional primary and booster doses | -287 (-1.4%) | -1,746 (-1.4%) | -7 (-0.4%) | -151 (-0.7%) |
| Expanding eligibility to high-risk children | -37 (-0.2%) | -319 (-0.3%) | -2 (-0.2%) | -40 (-0.2%) |
| Adults with comorbidities | | | | |
| **Prioritising within existing rollout capacity** | | | | |
| 25% primary dose only | -66 (-0.3%) | -96 (-0.1%) | -8 (-0.5%) | -71 (-0.3%) |
| 50% primary dose only | -86 (-0.4%) | -112 (-0.1%) | -11 (-0.7%) | -93 (-0.4%) |
| 75% primary dose only | -88 (-0.4%) | -106 (-0.1%) | -11 (-0.7%) | -96 (-0.4%) |
| 50% primary and booster provision | -4,677 (-22.1%) | -9,188 (-7.5%) | -517 (-34.1%) | -4,762 (-21.4%) |
| **Additional rollout capacity (opportunistic vaccination during regular care visits)** | | | | |
| Additional primary doses | -141 (-0.7%) | -432 (-0.4%) | -12 (-0.8%) | -134 (-0.6%) |
| Additional primary and booster doses | -5,156 (-24.3%) | -10,554 (-8.6%) | -560 (-37.0%) | -5,213 (-23.5%) |

**Table S4.10** Sensitivity analysis for prioritisation of high-risk groups where the individuals have **3 times the risk** of the general population. Severe outcomes applicable to the general population presented only.

|  | Incidence of severe disease | Hospitalisations | Deaths | YLL |
| --- | --- | --- | --- | --- |
| Pregnant women | | | | |
| **Prioritising within existing rollout capacity** | | | | |
| 25% primary dose only | 4 (0.0%) | -23 (0.0%) | 1 (0.1%) | 8 (0.0%) |
| 50% primary dose only | 5 (0.0%) | -21 (0.0%) | 1 (0.1%) | 9 (0.0%) |
| 75% primary dose only | 5 (0.0%) | -20 (0.0%) | 1 (0.1%) | 9 (0.0%) |
| 50% primary and booster provision | -373 (-1.8%) | -2,618 (-2.1%) | -1 (0.0%) | -134 (-0.6%) |
| **Additional rollout capacity (opportunistic vaccination during antenatal visits)** | | | | |
| Additional primary doses | -37 (-0.2%) | -178 (-0.1%) | -2 (-0.1%) | -29 (-0.1%) |
| Additional primary and booster doses | -491 (-2.3%) | -3,032 (-2.5%) | -9 (-0.6%) | -241 (-1.1%) |
| Expanding eligibility to high-risk children | -44 (-0.2%) | -494 (-0.4%) | -3 (-0.2%) | -53 (-0.2%) |
| Adults with comorbidities | | | | |
| **Prioritising within existing rollout capacity** | | | | |
| 25% primary dose only | -75 (-0.4%) | -126 (-0.1%) | -8 (-0.6%) | -76 (-0.3%) |
| 50% primary dose only | -99 (-0.5%) | -154 (-0.1%) | -11 (-0.7%) | -100 (-0.5%) |
| 75% primary dose only | -101 (-0.5%) | -151 (-0.1%) | -12 (-0.8%) | -103 (-0.5%) |
| 50% primary and booster provision | -5,139 (-24.2%) | -10,740 (-8.8%) | -528 (-34.8%) | -5,025 (-22.6%) |
| **Additional rollout capacity (opportunistic vaccination during regular care visits)** | | | | |
| Additional primary doses | -148 (-0.7%) | -457 (-0.4%) | -13 (-0.8%) | -138 (-0.6%) |
| Additional primary and booster doses | -5,644 (-26.6%) | -12,197 (-10.0%) | -572 (-37.8%) | -5,492 (-24.7%) |

##

## **S4.8 Influence of pre-existing infection-derived immunity**

Here, we provide sensitivity analysis for the influence of pre-existing infection-derived immunity on our model’s results. The absolute number of outcomes averted is inversely related to infection-derived immunity, that is, more outcomes are averted by a vaccine with lower infection-derived immunity in the initial state of the model (Table S4.11-12). While the absolute effect of prioritisation strategies changed, the relative effect changed only slightly, and subsequent qualitative conclusions did not change. This sensitivity analysis supports the robustness of our paper’s results irrespective of the model’s fit to reported cases.

**Table S4.11** *Sensitivity analysis using an initial state with 50% of fitted seroprevalence.* Cumulative outcomes prevented by prioritisation strategies including children 5 to 17 years. Outcomes prevented calculated relative to current adult vaccination program with a population coverage target of 51.6%. Red/green shading indicates the increase/reduction of an outcome.

|  | **Children (0-4 years)** | | | | | **Children (5-17 years)** | | | | | **Adults (18+ years)** | | | | | **Overall** | | | | |
| --- | --- | --- | --- | --- | --- | --- | --- | --- | --- | --- | --- | --- | --- | --- | --- | --- | --- | --- | --- | --- |
|  | Cases | Severe disease | Hospitalisations | Deaths | YLL | Cases | Severe disease | Hospitalisations | Deaths | YLL | Cases | Severe disease | Hospitalisations | Deaths | YLL | Cases | Severe disease | Hospitalisations | Deaths | YLL |
| **Expanding to children concurrently with the adult rollout** | | | | | | | | | | | | | | | | | | | | |
| 51.6% | 1305 | 0 | 18 | 0 | 2 | -90142 | -63 | -3785 | -6 | -336 | 75754 | 1546 | 4880 | 109 | 1284 | -13083 | 1483 | 1113 | 103 | 950 |
|  | 0.1% | 0.1% | 0.1% | 0.1% | 0.1% | -2.0% | -7.7% | -7.7% | -7.7% | -7.7% | 1.5% | 6.3% | 6.1% | 6.4% | 6.3% | -0.1% | 5.8% | 0.8% | 5.6% | 3.5% |
| 60% | -14432 | -2 | -163 | 0 | -19 | -158270 | -87 | -5214 | -9 | -463 | 3320 | 934 | 2994 | 64 | 767 | -169383 | 845 | -2382 | 55 | 285 |
|  | -0.9% | -0.9% | -0.9% | -0.9% | -0.9% | -3.6% | -10.6% | -10.6% | -10.6% | -10.6% | 0.1% | 3.8% | 3.8% | 3.7% | 3.8% | -1.5% | 3.3% | -1.6% | 3.0% | 1.1% |
| 70% | -48521 | -6 | -571 | -1 | -66 | -299494 | -119 | -7130 | -12 | -633 | -140936 | -2 | 70 | -4 | -18 | -488951 | -127 | -7631 | -18 | -718 |
|  | -3.2% | -3.1% | -3.1% | -3.1% | -3.1% | -6.8% | -14.5% | -14.5% | -14.5% | -14.5% | -2.7% | 0.0% | 0.1% | -0.3% | -0.1% | -4.4% | -0.5% | -5.2% | -1.0% | -2.7% |
| 75.5% | -59954 | -7 | -710 | -1 | -83 | -362618 | -130 | -7831 | -13 | -695 | -192745 | -309 | -899 | -27 | -275 | -615317 | -447 | -9440 | -41 | -1053 |
|  | -3.9% | -3.8% | -3.8% | -3.8% | -3.8% | -8.2% | -15.9% | -15.9% | -15.9% | -15.9% | -3.8% | -1.3% | -1.1% | -1.6% | -1.4% | -5.6% | -1.7% | -6.4% | -2.3% | -3.9% |
| **Expanding to children after adult rollout** | | | | | | | | | | | | | | | | | | | | |
| 60% | -16639 | -2 | -188 | 0 | -22 | -108939 | -51 | -3046 | -5 | -270 | -59893 | -326 | -954 | -26 | -285 | -185470 | -379 | -4187 | -32 | -577 |
|  | -1.1% | -1.0% | -1.0% | -1.0% | -1.0% | -2.5% | -6.2% | -6.2% | -6.2% | -6.2% | -1.2% | -1.3% | -1.2% | -1.5% | -1.4% | -1.7% | -1.5% | -2.8% | -1.7% | -2.2% |
| 70% | -47707 | -6 | -561 | -1 | -65 | -278793 | -93 | -5582 | -9 | -495 | -168863 | -899 | -2708 | -70 | -774 | -495362 | -998 | -8851 | -81 | -1335 |
|  | -3.1% | -3.0% | -3.0% | -3.0% | -3.0% | -6.3% | -11.4% | -11.4% | -11.4% | -11.4% | -3.3% | -3.6% | -3.4% | -4.1% | -3.8% | -4.5% | -3.9% | -6.0% | -4.4% | -5.0% |
| 75.5% | -57757 | -7 | -683 | -1 | -79 | -357006 | -108 | -6486 | -11 | -575 | -203162 | -1078 | -3260 | -84 | -928 | -617926 | -1193 | -10429 | -96 | -1583 |
|  | -3.8% | -3.7% | -3.7% | -3.7% | -3.7% | -8.1% | -13.2% | -13.2% | -13.2% | -13.2% | -4.0% | -4.4% | -4.1% | -4.9% | -4.6% | -5.6% | -4.6% | -7.1% | -5.3% | -5.9% |

**Table S4.12** *Sensitivity analysis using an initial state with 50% of fitted seroprevalence.* Cumulative outcomes prevented by risk-specific prioritisation strategies. Outcomes prevented calculated relative to current adult vaccination program with a population coverage target of 51.6% and uniform eligibility. Red/green shading indicates the increase/reduction of an outcome.

|  | Incidence | Incidence of severe disease | Hospitalisations | Deaths | YLL | Neonatal deaths |
| --- | --- | --- | --- | --- | --- | --- |
| Pregnant women | | | | | | |
| **Prioritising within existing rollout capacity** | | | | | | |
| 25% primary dose only | 810 (0.0%) | 19 (0.1%) | -34 (0.0%) | 3 (0.2%) | 28 (0.1%) | -92 (-2.4%) |
| 50% primary dose only | 1,176 (0.0%) | 21 (0.1%) | -36 (0.0%) | 3 (0.2%) | 30 (0.1%) | -101 (-2.6%) |
| 75% primary dose only | 1,311 (0.0%) | 22 (0.1%) | -36 (0.0%) | 3 (0.2%) | 31 (0.1%) | -103 (-2.7%) |
| 50% primary and booster provision | -28,488 (-0.3%) | -212 (-0.8%) | -2,040 (-1.4%) | 9 (0.5%) | -16 (-0.1%) | -1,339 (-34.8%) |
| **Additional rollout capacity (antenatal visits)** | | | | | | |
| Additional primary doses | -2,838 (0.0%) | -74 (-0.3%) | -310 (-0.2%) | -4 (-0.2%) | -53 (-0.2%) | -85 (-2.2%) |
| Additional primary and booster doses | -34,315 (-0.3%) | -463 (-1.8%) | -2,728 (-1.9%) | -11 (-0.6%) | -236 (-0.9%) | -1,293 (-33.6%) |
| Expanding eligibility to high-risk children | -5,471 (0.0%) | -76 (-0.3%) | -559 (-0.4%) | -4 (-0.2%) | -71 (-0.3%) | -233 (-6.0%) |
| Adults with comorbidities | | | | | | |
| **Prioritising within existing rollout capacity** | | | | | | |
| 25% primary dose only | 889 (0.0%) | -223 (-0.9%) | -383 (-0.3%) | -27 (-1.5%) | -236 (-0.9%) | NA |
| 50% primary dose only | 2,882 (0.0%) | -348 (-1.4%) | -581 (-0.4%) | -42 (-2.3%) | -369 (-1.4%) | NA |
| 75% primary dose only | 3,931 (0.0%) | -374 (-1.5%) | -616 (-0.4%) | -45 (-2.5%) | -397 (-1.5%) | NA |
| 50% primary and booster provision | -54,966 (-0.5%) | -4,873 (-19.0%) | -9,781 (-6.6%) | -529 (-28.9%) | -4,918 (-18.3%) | NA |
| **Additional rollout capacity** | | | | | | |
| Additional primary doses | -6,737 (-0.1%) | -437 (-1.7%) | -1,049 (-0.7%) | -42 (-2.3%) | -417 (-1.6%) | NA |
| Additional primary and booster doses | -64,563 (-0.6%) | -5,794 (-22.6%) | -12,079 (-8.2%) | -612 (-33.4%) | -5,763 (-21.5%) | NA |

**Table S4.13** *Sensitivity analysis using an initial state with 150% of fitted seroprevalence.* Cumulative outcomes prevented by prioritisation strategies including children 5 to 17 years. Outcomes prevented calculated relative to current adult vaccination program with a population coverage target of 51.6%. Red/green shading indicates the increase/reduction of an outcome.

|  | **Children (0-4 years)** | | | | | **Children (5-17 years)** | | | | | **Adults (18+ years)** | | | | | **Overall** | | | | |
| --- | --- | --- | --- | --- | --- | --- | --- | --- | --- | --- | --- | --- | --- | --- | --- | --- | --- | --- | --- | --- |
|  | Cases | Severe disease | Hospitalisations | Deaths | YLL | Cases | Severe disease | Hospitalisations | Deaths | YLL | Cases | Severe disease | Hospitalisations | Deaths | YLL | Cases | Severe disease | Hospitalisations | Deaths | YLL |
| **Expanding to children concurrently with the adult rollout** | | | | | | | | | | | | | | | | | | | | |
| 51.6% | 2025 | 0 | 28 | 0 | 3 | -62906 | -61 | -3679 | -6 | -327 | 34116 | 1148 | 3710 | 79 | 943 | -26764 | 1087 | 59 | 73 | 619 |
|  | 0.2% | 0.2% | 0.2% | 0.2% | 0.2% | -2.1% | -11.1% | -11.1% | -11.0% | -11.0% | 1.0% | 6.9% | 6.9% | 6.8% | 6.8% | -0.4% | 6.2% | 0.1% | 5.8% | 3.4% |
| 60% | -4191 | 0 | -39 | 0 | -5 | -115338 | -84 | -5044 | -9 | -448 | -13016 | 603 | 2057 | 37 | 478 | -132545 | 519 | -3027 | 29 | 25 |
|  | -0.4% | -0.3% | -0.3% | -0.3% | -0.3% | -3.8% | -15.2% | -15.2% | -15.1% | -15.1% | -0.4% | 3.6% | 3.8% | 3.2% | 3.5% | -1.7% | 3.0% | -3.0% | 2.3% | 0.1% |
| 70% | -28606 | -3 | -329 | -1 | -38 | -232440 | -110 | -6624 | -11 | -588 | -127014 | -180 | -361 | -21 | -183 | -388059 | -294 | -7314 | -32 | -809 |
|  | -2.8% | -2.6% | -2.6% | -2.6% | -2.6% | -7.7% | -19.9% | -19.9% | -19.9% | -19.9% | -3.6% | -1.1% | -0.7% | -1.8% | -1.3% | -5.1% | -1.7% | -7.3% | -2.6% | -4.4% |
| 75.5% | -36996 | -4 | -430 | -1 | -50 | -281308 | -119 | -7154 | -12 | -635 | -166043 | -411 | -1090 | -37 | -377 | -484347 | -535 | -8674 | -50 | -1062 |
|  | -3.6% | -3.4% | -3.4% | -3.4% | -3.4% | -9.4% | -21.5% | -21.5% | -21.5% | -21.5% | -4.7% | -2.5% | -2.0% | -3.2% | -2.7% | -6.4% | -3.1% | -8.7% | -4.0% | -5.8% |
| **Expanding to children after adult rollout** | | | | | | | | | | | | | | | | | | | | |
| 60% | -4540 | 0 | -45 | 0 | -5 | -100022 | -54 | -3216 | -5 | -286 | -16639 | -121 | -294 | -12 | -117 | -121200 | -175 | -3556 | -18 | -408 |
|  | -0.4% | -0.4% | -0.4% | -0.4% | -0.4% | -3.3% | -9.7% | -9.7% | -9.7% | -9.7% | -0.5% | -0.7% | -0.5% | -1.1% | -0.8% | -1.6% | -1.0% | -3.6% | -1.4% | -2.2% |
| 70% | -26539 | -3 | -307 | -1 | -36 | -247395 | -89 | -5363 | -9 | -476 | -93553 | -535 | -1543 | -45 | -475 | -367487 | -627 | -7212 | -55 | -987 |
|  | -2.6% | -2.4% | -2.4% | -2.4% | -2.4% | -8.2% | -16.1% | -16.1% | -16.1% | -16.1% | -2.6% | -3.2% | -2.9% | -3.9% | -3.4% | -4.9% | -3.6% | -7.2% | -4.4% | -5.4% |
| 75.5% | -33966 | -4 | -396 | -1 | -46 | -308906 | -101 | -6055 | -10 | -538 | -118786 | -666 | -1948 | -55 | -588 | -461658 | -771 | -8400 | -66 | -1172 |
|  | -3.3% | -3.1% | -3.1% | -3.1% | -3.1% | -10.3% | -18.2% | -18.2% | -18.2% | -18.2% | -3.4% | -4.0% | -3.6% | -4.8% | -4.3% | -6.1% | -4.4% | -8.4% | -5.3% | -6.4% |

**Table S4.14** *Sensitivity analysis using an initial state with 150% of fitted seroprevalence.* Cumulative outcomes prevented by risk-specific prioritisation strategies. Outcomes prevented calculated relative to current adult vaccination program with a population coverage target of 51.6% and uniform eligibility. Red/green shading indicates the increase/reduction of an outcome.

|  | Incidence | Incidence of severe disease | Hospitalisations | Deaths | YLL | Neonatal deaths |
| --- | --- | --- | --- | --- | --- | --- |
| Pregnant women | | | | | | |
| **Prioritising within existing rollout capacity** | | | | | | |
| 25% primary dose only | 578 (0.0%) | -1 (0.0%) | 5 (0.0%) | 0 (0.0%) | -1 (0.0%) | 6 (0.2%) |
| 50% primary dose only | 851 (0.0%) | 0 (0.0%) | 9 (0.0%) | 0 (0.0%) | 0 (0.0%) | 6 (0.2%) |
| 75% primary dose only | 956 (0.0%) | 0 (0.0%) | 10 (0.0%) | 0 (0.0%) | 0 (0.0%) | 6 (0.2%) |
| 50% primary and booster provision | -21,027 (-0.3%) | -368 (-2.1%) | -2,211 (-2.2%) | -8 (-0.6%) | -180 (-1.0%) | -1,079 (-41.3%) |
| **Additional rollout capacity (antenatal visits)** | | | | | | |
| Additional primary doses | -231 (0.0%) | 5 (0.0%) | -8 (0.0%) | 0 (0.0%) | 2 (0.0%) | 5 (0.2%) |
| Additional primary and booster doses | -21,295 (-0.3%) | -341 (-2.0%) | -2,222 (-2.2%) | -5 (-0.4%) | -162 (-0.9%) | -1,081 (-41.4%) |
| Expanding eligibility to high-risk children | -2,753 (0.0%) | -1 (0.0%) | -227 (-0.2%) | 0 (0.0%) | -15 (-0.1%) | -115 (-4.4%) |
| Adults with comorbidities | | | | | | |
| **Prioritising within existing rollout capacity** | | | | | | |
| 25% primary dose only | 806 (0.0%) | 25 (0.1%) | 53 (0.1%) | 3 (0.2%) | 27 (0.1%) | NA |
| 50% primary dose only | 1,826 (0.0%) | 35 (0.2%) | 81 (0.1%) | 4 (0.3%) | 38 (0.2%) | NA |
| 75% primary dose only | 2,400 (0.0%) | 38 (0.2%) | 91 (0.1%) | 4 (0.4%) | 41 (0.2%) | NA |
| 50% primary and booster provision | -39,333 (-0.5%) | -4,671 (-26.8%) | -9,538 (-9.6%) | -497 (-39.9%) | -4,659 (-25.6%) | NA |
| **Additional rollout capacity** | | | | | | |
| Additional primary doses | -486 (0.0%) | 36 (0.2%) | 26 (0.0%) | 4 (0.3%) | 32 (0.2%) | NA |
| Additional primary and booster doses | -34,774 (-0.5%) | -4,688 (-26.9%) | -9,704 (-9.7%) | -499 (-40.1%) | -4,690 (-25.8%) | NA |

# **Reference List**

1. Bubar KM, Reinholt K, Kissler SM, Lipsitch M, Cobey S, Grad YH, Larremore DB: **Model-informed COVID-19 vaccine prioritization strategies by age and serostatus**. *Science* 2021, **371**(6532):916-921.

2. Buckner JH, Chowell G, Springborn MR: **Dynamic prioritization of COVID-19 vaccines when social distancing is limited for essential workers**. *Proc Natl Acad Sci U S A* 2021, **118**(16).

3. Choi W, Shim E: **Vaccine Effects on Susceptibility and Symptomatology Can Change the Optimal Allocation of COVID-19 Vaccines: South Korea as an Example**. *J Clin Med* 2021, **10**(13).

4. Gozzi N, Bajardi P, Perra N: **The importance of non-pharmaceutical interventions during the COVID-19 vaccine rollout**. *PLoS Comput Biol* 2021, **17**(9):e1009346.

5. Sunohara S, Asakura T, Kimura T, Ozawa S, Oshima S, Yamauchi D, Tamakoshi A: **Effective vaccine allocation strategies, balancing economy with infection control against COVID-19 in Japan**. *PLoS One* 2021, **16**(9):e0257107.

6. Liu Y, Gayle AA, Wilder-Smith A, Rocklov J: **The reproductive number of COVID-19 is higher compared to SARS coronavirus**. *J Travel Med* 2020, **27**(2).

7. Liu Y, Rocklov J: **The reproductive number of the Delta variant of SARS-CoV-2 is far higher compared to the ancestral SARS-CoV-2 virus**. *J Travel Med* 2021, **28**(7).

8. Davies NG, Klepac P, Liu Y, Prem K, Jit M, group CC-w, Eggo RM: **Age-dependent effects in the transmission and control of COVID-19 epidemics**. *Nat Med* 2020, **26**(8):1205-1211.

9. Prem K, Zandvoort KV, Klepac P, Eggo RM, Davies NG, Centre for the Mathematical Modelling of Infectious Diseases C-WG, Cook AR, Jit M: **Projecting contact matrices in 177 geographical regions: An update and comparison with empirical data for the COVID-19 era**. *PLoS Comput Biol* 2021, **17**(7):e1009098.

10. International Vaccine Access Center, World Health Organisation: **Results of COVID-19 Vaccine Effectiveness Studies: An Ongoing Systematic Review**. In*.*; 2022.

11. Kirsebom FCM, Andrews N, Stowe J, Toffa S, Sachdeva R, Gallagher E, Groves N, O'Connell AM, Chand M, Ramsay M *et al*: **COVID-19 vaccine effectiveness against the omicron (BA.2) variant in England**. *Lancet Infect Dis* 2022, **22**(7):931-933.

12. Altarawneh HN, Chemaitelly H, Ayoub HH, Tang P, Hasan MR, Yassine HM, Al-Khatib HA, Smatti MK, Coyle P, Al-Kanaani Z *et al*: **Effects of Previous Infection and Vaccination on Symptomatic Omicron Infections**. *New England Journal of Medicine* 2022, **387**(1):21-34.

13. Bekker LG, Garrett N, Goga A, Fairall L, Reddy T, Yende-Zuma N, Kassanjee R, Collie S, Sanne I, Boulle A *et al*: **Effectiveness of the Ad26.COV2.S vaccine in health-care workers in South Africa (the Sisonke study): results from a single-arm, open-label, phase 3B, implementation study**. *Lancet* 2022, **399**(10330):1141-1153.

14. Andrews N, Tessier E, Stowe J, Gower C, Kirsebom F, Simmons R, Gallagher E, Thelwall S, Groves N, Dabrera G *et al*: **Duration of Protection against Mild and Severe Disease by Covid-19 Vaccines**. *N Engl J Med* 2022, **386**(4):340-350.

15. Bruxvoort KJ, Sy LS, Qian L, Ackerson BK, Luo Y, Lee GS, Tian Y, Florea A, Aragones M, Tubert JE *et al*: **Effectiveness of mRNA-1273 against delta, mu, and other emerging variants of SARS-CoV-2: test negative case-control study**. *BMJ* 2021, **375**:e068848.

16. Suah JL, Husin M, Tok PSK, Tng BH, Thevananthan T, Low EV, Appannan MR, Muhamad Zin F, Mohd Zin S, Yahaya H *et al*: **Waning COVID-19 Vaccine Effectiveness for BNT162b2 and CoronaVac in Malaysia: An Observational Study**. *Int J Infect Dis* 2022, **119**:69-76.

17. Rearte A, Castelli JM, Rearte R, Fuentes N, Pennini V, Pesce M, Barbeira PB, Iummato LE, Laurora M, Bartolomeu ML *et al*: **Effectiveness of rAd26-rAd5, ChAdOx1 nCoV-19, and BBIBP-CorV vaccines for risk of infection with SARS-CoV-2 and death due to COVID-19 in people older than 60 years in Argentina: a test-negative, case-control, and retrospective longitudinal study**. *The Lancet* 2022, **399**(10331):1254-1264.

18. Belayachi J, Obtel M, Razine R, Abouqal R: **Long term effectiveness of inactivated vaccine BBIBP-CorV (Vero Cells) against COVID-19 associated severe and critical hospitalization in Morocco**. *medRxiv* 2022:2022.2001.2025.22269822.

19. Voko Z, Kiss Z, Surjan G, Surjan O, Barcza Z, Wittmann I, Molnar GA, Nagy D, Muller V, Bogos K *et al*: **Effectiveness and Waning of Protection With Different SARS-CoV-2 Primary and Booster Vaccines During the Delta Pandemic Wave in 2021 in Hungary (HUN-VE 3 Study)**. *Front Immunol* 2022, **13**:919408.

20. Polinski J, Weckstein A, Batech M, Kabelac C, Kamath T, Harvey R, Jain S, Rassen J, Khan N, Schneeweiss S: **Durability of the Single-Dose Ad26.COV2.S Vaccine in the Prevention of COVID-19 Infections and Hospitalizations in the US Before and During the Delta Variant Surge**. *JAMA Network Open* 2022, **5**:222959.

21. Nasreen S, Chung H, He S, Brown KA, Gubbay JB, Buchan SA, Fell DB, Austin PC, Schwartz KL, Sundaram ME *et al*: **Effectiveness of COVID-19 vaccines against symptomatic SARS-CoV-2 infection and severe outcomes with variants of concern in Ontario**. *Nature Microbiology* 2022, **7**(3):379-385.

22. Florea A, Sy LS, Luo Y, Qian L, Bruxvoort KJ, Ackerson BK, Lee GS, Ku JH, Tubert JE, Tian Y *et al*: **Durability of mRNA-1273 against COVID-19 in the time of Delta: Interim results from an observational cohort study**. *PLOS ONE* 2022, **17**(4):e0267824.

23. Pouwels KB, Pritchard E, Matthews PC, Stoesser N, Eyre DW, Vihta K-D, House T, Hay J, Bell JI, Newton JN *et al*: **Effect of Delta variant on viral burden and vaccine effectiveness against new SARS-CoV-2 infections in the UK**. *Nature Medicine* 2021, **27**(12):2127-2135.

24. Natarajan K, Prasad N, Dascomb K, Irving SA, Yang DH, Gaglani M, Klein NP, DeSilva MB, Ong TC, Grannis SJ *et al*: **Effectiveness of Homologous and Heterologous COVID-19 Booster Doses Following 1 Ad.26.COV2.S (Janssen [Johnson & Johnson]) Vaccine Dose Against COVID-19-Associated Emergency Department and Urgent Care Encounters and Hospitalizations Among Adults - VISION Network, 10 States, December 2021-March 2022**. *MMWR Morb Mortal Wkly Rep* 2022, **71**(13):495-502.

25. Gram MA, Emborg H-D, Schelde AB, Friis NU, Nielsen KF, Moustsen-Helms IR, Legarth R, Lam JUH, Chaine M, Malik AZ *et al*: **Vaccine effectiveness against SARS-CoV-2 infection or COVID-19 hospitalization with the Alpha, Delta, or Omicron SARS-CoV-2 variant: A nationwide Danish cohort study**. *PLOS Medicine* 2022, **19**(9):e1003992.

26. Ranzani OT, Hitchings MDT, de Melo RL, de Franca GVA, Fernandes CFR, Lind ML, Torres MSS, Tsuha DH, David LCS, Said RFC *et al*: **Effectiveness of an inactivated Covid-19 vaccine with homologous and heterologous boosters against Omicron in Brazil**. *Nat Commun* 2022, **13**(1):5536.

27. Baum U, Poukka E, Leino T, Kilpi T, Nohynek H, Palmu AA: **High vaccine effectiveness against severe COVID-19 in the elderly in Finland before and after the emergence of Omicron**. *BMC Infect Dis* 2022, **22**(1):816.

28. Florentino PTV, Millington T, Cerqueira-Silva T, Robertson C, de Araújo Oliveira V, Júnior JBS, Alves FJO, Penna GO, Vital Katikireddi S, Boaventura VS *et al*: **Vaccine effectiveness of two-dose BNT162b2 against symptomatic and severe COVID-19 among adolescents in Brazil and Scotland over time: a test-negative case-control study**. *The Lancet Infectious Diseases* 2022, **22**(11):1577-1586.

29. Monge S, Rojas-Benedicto A, Olmedo C, Mazagatos C, José Sierra M, Limia A, Martín-Merino E, Larrauri A, Hernán MA: **Effectiveness of mRNA vaccine boosters against infection with the SARS-CoV-2 omicron (B.1.1.529) variant in Spain: a nationwide cohort study**. *Lancet Infect Dis* 2022, **22**(9):1313-1320.

30. Cerqueira-Silva T, de Araujo Oliveira V, Paixao ES, Junior JB, Penna GO, Werneck GL, Pearce N, Barreto ML, Boaventura VS, Barral-Netto M: **Duration of protection of CoronaVac plus heterologous BNT162b2 booster in the Omicron period in Brazil**. *Nat Commun* 2022, **13**(1):4154.

31. Cerqueira-Silva T, Shah SA, Robertson C, Sanchez MN, Katikireddi SV, de Araújo Oliveira V, Paixão ES, Rudan I, Júnior JB, Penna GO *et al*: **Waning of mRNA Boosters after Homologous Primary Series with BNT162b2 or ChadOx1 Against Symptomatic Infection and Severe COVID-19 in Brazil and Scotland: A Test-Negative Design Case-Control Study**. *SSRN Electronic Journal* 2022.

32. Stowe J, Andrews N, Kirsebom F, Ramsay M, Bernal JL: **Effectiveness of COVID-19 vaccines against Omicron and Delta hospitalisation, a test negative case-control study**. *Nat Commun* 2022, **13**(1):5736.

33. Smid M, Berec L, Pribylova L, Majek O, Pavlik T, Jarkovsky J, Weiner J, Barusova T, Trnka J: **Protection by Vaccines and Previous Infection Against the Omicron Variant of Severe Acute Respiratory Syndrome Coronavirus 2**. *J Infect Dis* 2022, **226**(8):1385-1390.

34. Andrews N, Stowe J, Kirsebom F, Toffa S, Rickeard T, Gallagher E, Gower C, Kall M, Groves N, O'Connell AM *et al*: **Covid-19 Vaccine Effectiveness against the Omicron (B.1.1.529) Variant**. *N Engl J Med* 2022, **386**(16):1532-1546.

35. Cerqueira-Silva T, Katikireddi SV, de Araujo Oliveira V, Flores-Ortiz R, Junior JB, Paixao ES, Robertson C, Penna GO, Werneck GL, Barreto ML *et al*: **Vaccine effectiveness of heterologous CoronaVac plus BNT162b2 in Brazil**. *Nat Med* 2022, **28**(4):838-843.

36. Chemaitelly H, Ayoub HH, Coyle P, Tang P, Yassine HM, Al-Khatib HA, Smatti MK, Hasan MR, Al-Kanaani Z, Al-Kuwari E *et al*: **Protection of Omicron sub-lineage infection against reinfection with another Omicron sub-lineage**. *Nat Commun* 2022, **13**(1):4675.

37. Chemaitelly H, Nagelkerke N, Ayoub HH, Coyle P, Tang P, Yassine HM, Al-Khatib HA, Smatti MK, Hasan MR, Al-Kanaani Z *et al*: **Duration of immune protection of SARS-CoV-2 natural infection against reinfection**. *Journal of Travel Medicine* 2022.

38. Khare S, Gurry C, Freitas L, Schultz MB, Bach G, Diallo A, Akite N, Ho J, Lee RT, Yeo W *et al*: **GISAID's Role in Pandemic Response**. *China CDC Wkly* 2021, **3**(49):1049-1051.

39. Pilz S, Theiler-Schwetz V, Trummer C, Krause R, Ioannidis JPA: **SARS-CoV-2 reinfections: Overview of efficacy and duration of natural and hybrid immunity**. *Environ Res* 2022, **209**:112911.

40. Altarawneh HN, Chemaitelly H, Hasan MR, Ayoub HH, Qassim S, AlMukdad S, Coyle P, Yassine HM, Al-Khatib HA, Benslimane FM *et al*: **Protection against the Omicron Variant from Previous SARS-CoV-2 Infection**. *N Engl J Med* 2022, **386**(13):1288-1290.

41. Cerqueira-Silva T, de Araujo Oliveira V, Paixao ES, Florentino PTV, Penna GO, Pearce N, Werneck GL, Barreto ML, Boaventura VS, Barral-Netto M: **Vaccination plus previous infection: protection during the omicron wave in Brazil**. *Lancet Infect Dis* 2022, **22**(7):945-946.

42. Ayoub HH, Mumtaz GR, Seedat S, Makhoul M, Chemaitelly H, Abu-Raddad LJ: **Estimates of global SARS-CoV-2 infection exposure, infection morbidity, and infection mortality rates in 2020**. *Glob Epidemiol* 2021, **3**:100068.

43. Seedat S, Chemaitelly H, Ayoub HH, Makhoul M, Mumtaz GR, Al Kanaani Z, Al Khal A, Al Kuwari E, Butt AA, Coyle P *et al*: **SARS-CoV-2 infection hospitalization, severity, criticality, and fatality rates in Qatar**. *Sci Rep* 2021, **11**(1):18182.

44. Ferguson N, Ghani A, Hinsley W, Volz E: **Report 50 - Hospitalisation risk for Omicron cases in England**. In: *Imperial College London.* Edited by London IC, vol. 22-12-2021: Imperial College London; 2021.

45. Lin L, Liu Y, Tang X, He D: **The Disease Severity and Clinical Outcomes of the SARS-CoV-2 Variants of Concern**. *Front Public Health* 2021, **9**:775224.

46. United Nations: **World Population Prospects 2019**. In*.* Online Edition; 2019.

47. World Bank Group: **Sierra Leone - COVID-19 Emergency Preparedness and Response Project (English)**. In*.* Washington, D.C.; 2021.

48. Treskova-Schwarzbach M, Haas L, Reda S, Pilic A, Borodova A, Karimi K, Koch J, Nygren T, Scholz S, Schonfeld V *et al*: **Pre-existing health conditions and severe COVID-19 outcomes: an umbrella review approach and meta-analysis of global evidence**. *BMC Med* 2021, **19**(1):212.

49. Feikin DR, Abu-Raddad LJ, Andrews N, Davies MA, Higdon MM, Orenstein WA, Patel MK: **Assessing vaccine effectiveness against severe COVID-19 disease caused by omicron variant. Report from a meeting of the World Health Organization**. *Vaccine* 2022, **40**(26):3516-3527.

50. Sierra Leone Noncommunicable Disease and Injuries Poverty Commission: **Sierra Leone Non-communicable Disease and Injuries Poverty Commission: Findings and Recommendations**. In*.*; 2020.

51. Odland ML, Bockarie T, Wurie H, Ansumana R, Lamin J, Nugent R, Bakolis I, Witham M, Davies J: **Prevalence and access to care for cardiovascular risk factors in older people in Sierra Leone: a cross-sectional survey**. *BMJ Open* 2020, **10**(9):e038520.

52. Statistics Sierra Leone & ICF: **Sierra Leone Demographic Health Survey 2019**. In*.* Freetown, Sierra Leone, and Rockville, Maryland, USA; 2020.

53. Elkasabi M: **DHS.rates: Calculates Demographic Indicators**. In: *R package version 0 91.* vol. R package version 0. 9.1. https://CRAN.R-project.org/package=DHS.rates; 2021.

54. **Guide to DHS Statistics** [https://dhsprogram.com/data/Guide-to-DHS-Statistics/index.htm#t=Guide_to_DHS_Statistics_DHS-7.htm]

55. **Coronavirus (COVID-19), infection in pregnancy** [https://www.rcog.org.uk/media/xsubnsma/2022-03-07-coronavirus-covid-19-infection-in-pregnancy-v15.pdf]

56. Carshon-Marsh R, Aimone A, Ansumana R, Swaray IB, Assalif A, Musa A, Meh C, Smart F, Hang Fu S, Newcombe L *et al*: **Child, maternal, and adult mortality in Sierra Leone: nationally representative mortality survey 2018–20**. *The Lancet Global Health* 2022, **10**(1):e114-e123.

57. Barrie MB, Lakoh S, Kelly JD, Kanu JS, Squire JS, Koroma Z, Bah S, Sankoh O, Brima A, Ansumana R *et al*: **SARS-CoV-2 antibody prevalence in Sierra Leone, March 2021: a cross-sectional, nationally representative, age-stratified serosurvey**. *BMJ Glob Health* 2021, **6**(11):e007271.

58. Barber RM, Sorensen RJD, Pigott DM, Bisignano C, Carter A, Amlag JO, Collins JK, Abbafati C, Adolph C, Allorant A *et al*: **Estimating global, regional, and national daily and cumulative infections with SARS-CoV-2 through Nov 14, 2021: a statistical analysis**. *Lancet* 2022, **399**(10344):2351-2380.

59. World Health Organization: **COVID-19 weekly epidemiological update, edition 82, 8 March 2022**. In*.* Geneva: World Health Organization; 2022.

60. Antia R, Halloran ME: **Transition to endemicity: Understanding COVID-19**. *Immunity* 2021, **54**(10):2172-2176.

61. Al-Tawfiq JA, Chu DT, Hoang VT, Memish ZA: **From Pandemicity to Endemicity: The Journey of SARS-CoV-2**. *J Epidemiol Glob Health* 2022, **12**(2):147-149.

62. Skjefte M, Ngirbabul M, Akeju O, Escudero D, Hernandez-Diaz S, Wyszynski DF, Wu JW: **COVID-19 vaccine acceptance among pregnant women and mothers of young children: results of a survey in 16 countries**. *Eur J Epidemiol* 2021, **36**(2):197-211.

63. Riley LE, Jamieson DJ: **Inclusion of Pregnant and Lactating Persons in COVID-19 Vaccination Efforts**. *Ann Intern Med* 2021, **174**(5):701-702.

64. Beigi RH, Krubiner C, Jamieson DJ, Lyerly AD, Hughes B, Riley L, Faden R, Karron R: **The need for inclusion of pregnant women in COVID-19 vaccine trials**. *Vaccine* 2021, **39**(6):868-870.

65. Prasad S, Kalafat E, Blakeway H, Townsend R, O'Brien P, Morris E, Draycott T, Thangaratinam S, Le Doare K, Ladhani S *et al*: **Systematic review and meta-analysis of the effectiveness and perinatal outcomes of COVID-19 vaccination in pregnancy**. *Nat Commun* 2022, **13**(1):2414.

66. Tenforde MW, Self WH, Zhu Y, Naioti EA, Gaglani M, Ginde AA, Jensen K, Talbot HK, Casey JD, Mohr NM *et al*: **Protection of mRNA vaccines against hospitalized COVID-19 in adults over the first year following authorization in the United States**. *Clin Infect Dis* 2022.

67. Kirsebom F, Andrews N, Sachdeva R, Stowe J, Ramsay M, Bernal JL: **Effectiveness of ChAdOx1-S COVID-19 Booster Vaccination against the Omicron and Delta variants in England**. *medRxiv* 2022:2022.2004.2029.22274483.

68. Lewis NM, Naioti EA, Self WH, Ginde AA, Douin DJ, Keipp Talbot H, Casey JD, Mohr NM, Zepeski A, Gaglani M *et al*: **Effectiveness of mRNA Vaccines Against COVID-19 Hospitalization by Age and Chronic Medical Conditions Burden Among Immunocompetent US Adults, March-August 2021**. *J Infect Dis* 2022, **225**(10):1694-1700.

69. Kim SS, Chung JR, Talbot HK, Grijalva CG, Wernli KJ, Kiniry E, Martin ET, Monto AS, Belongia EA, McLean HQ *et al*: **Effectiveness of two and three mRNA COVID-19 vaccine doses against Omicron- and Delta-Related outpatient illness among adults, October 2021–February 2022**. *Influenza and Other Respiratory Viruses* 2022, **16**(6):975-985.
